# Supplementary material for: Lasianosides F–I: A New Iridoid and Three New Bis-Iridoid Glycosides from the Leaves of Lasianthus verticillatus (Lour.) Merr
Source: Molecules. 2020 Jun 17;25(12):2798. doi: 10.3390/molecules25122798 (PMC7356845; doi:10.3390/molecules25122798)
Supplement: Supplementary file 1 [file molecules-25-02798-s001.zip › Supplementary file/Supplementary data.pdf]

Supplementary material

Lasianosides F-I: A new iridoid and three new bis-iridoid glycosides from the leaves of *Lasianthus verticillatus* (Lour.) Merr.

Gadah Abdulaziz Al-Hamoud[1], Raha Saud Orfali[1], Yoshio Takeda[2], Sachiko Sugimoto[3], Yoshi Yamano[3], Nawal M. Al Musayeib[1], Omer Ibrahim Fantoukh[1], Musarat Amina[1], Hideaki Otsuka[4] and Katsuyoshi Matsunami[3,*]

[1] Department of Pharmacognosy, College of Pharmacy, King Saud University, Riyadh 11495, Kingdom of Saudi Arabia; galhamoud@ksu.edu.sa (G.A.A.); rorfali@ksu.edu.sa (R.S.O.); nalmusayeib@ksu.edu.sa (N. M. A.); ofantoukh@ksu.edu.sa (O.I.F.); mamina@ksu.edu.sa (M. A.)

[2] Faculty of Integrated Arts and Sciences, The University of Tokushima, 1-1 Minamijosanjima-Cho, Tokushima 770-8502, Japan; takeda@ias.tokushima-u.ac.jp (Y.T.)

[3] Graduate School of Biomedical and Health Sciences, Hiroshima University, 1-2-3 Kasumi, Minami-ku, Hiroshima 734-8553, Japan; matunamil@hiroshima-u.ac.jp (K.M.); ssugimot@hiroshima-u.ac.jp (S.S.); yamano@hiroshima-u.ac.jp (Y.Y.)

[4] Faculty of Pharmacy, Yasuda Women's University, 6-13-1 Yasuhigashi, Asaminami-ku, Hiroshima 731-0153, Japan; otsuka-h@yasuda-u.ac.jp (H.O.)

Contents:

Figure S1. The HRESIMS spectrum of **1**.
Figure S2. The ^{1}H NMR spectrum of **1** in CD_3OD, 500 MHz.
Figure S3. The ^{13}C NMR spectrum of **1** in CD_3OD, 175 MHz.
Figure S4. The DEPT-135 NMR spectrum of **1** in CD_3OD, 175 MHz.
Figure S5. The COSY spectrum of **1** in CD_3OD, 500 MHz.
Figure S6. The HSQC spectrum of **1** in CD_3OD, 700 MHz.
Figure S7. The HMBC spectrum of **1** in CD_3OD, 700 MHz.
Figure S8. The NOESY spectrum of **1** in CD_3OD, 500 MHz.
Figure S9. The UV spectrum of **1**.
Figure S10. The IR spectrum of **1**.
Figure S11. The HRESIMS spectrum of **2**.
Figure S12. The ^{1}H NMR spectrum of **2** in CD_3OD, 500 MHz.
Figure S13. The ^{13}C NMR spectrum of **2** in CD_3OD, 125 MHz.
Figure S14. The ^{13}C NMR spectrum of **2** in CD_3OD, 125 MHz (magnified)
Figure S15. The DEPT-135 NMR spectrum of **2** in CD_3OD, 175 MHz.
Figure S16. The COSY spectrum of **2** in CD_3OD, 500 MHz.
Figure S17.. The HSQC spectrum of **2** in CD_3OD, 700 MHz.
Figure S18. The HMBC spectrum of **2** in CD_3OD, 700 MHz.
Figure S19. The NOESY spectrum of **2** in CD_3OD, 500 MHz.
Figure S20. The UV spectrum of **2**.
Figure S21. The IR spectrum of **2**.
Figure S22. The HRESIMS spectrum of **3**.
Figure S23. The ^{1}H NMR spectrum of **3** in CD_3OD, 500 MHz.
Figure S24. The ^{13}C NMR spectrum of **3** in CD_3OD, 175 MHz.
Figure S25. The DEPT-135 NMR spectrum of **3** in CD_3OD, 175 MHz.
Figure S26. The COSY spectrum of **3** in CD_3OD, 500 MHz.
Figure S27. The HSQC spectrum of **3** in CD_3OD, 700 MHz.
Figure S28. The HMBC spectrum of **3** in CD_3OD, 700 MHz.
Figure S29. The NOESY spectrum of **3** in CD_3OD, 500 MHz.
Figure S30. The UV spectrum of **3**.
Figure S31. The IR spectrum of **3**.
Figure S32. The HRESIMS spectrum of **4**.
Figure S33. The ^{1}H NMR spectrum of **4** in CD_3OD, 500 MHz.
Figure S34. The ^{13}C NMR spectrum of **4** in CD_3OD, 175 MHz.
Figure S35. The DEPT-135 NMR spectrum of **4** in CD_3OD, 175 MHz.
Figure S36. The COSY spectrum of **4** in CD_3OD, 500 MHz.
Figure S37 The HSQC spectrum of **4** in CD_3OD, 700 MHz.
Figure S38. The HMBC spectrum of **4** in CD_3OD, 700 MHz.
Figure S39. The NOESY spectrum of **4** in CD_3OD, 500 MHz.
Figure S40. The UV spectrum of **4**.
Figure S41. The IR spectrum of **4**.

Figure S1. The HRESIMS spectrum of **1**.

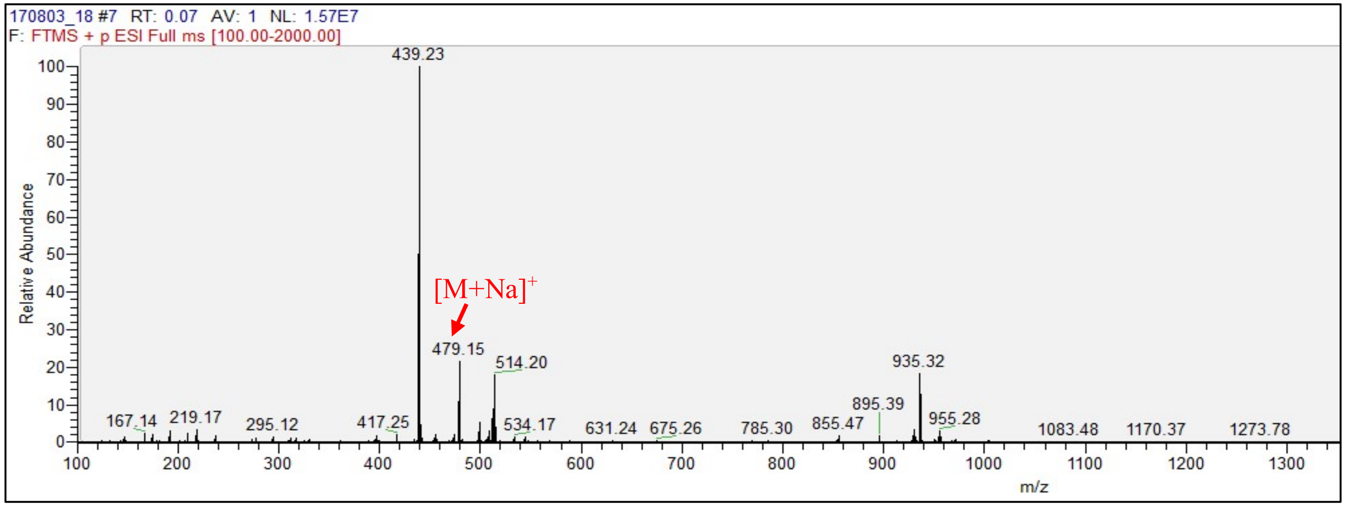

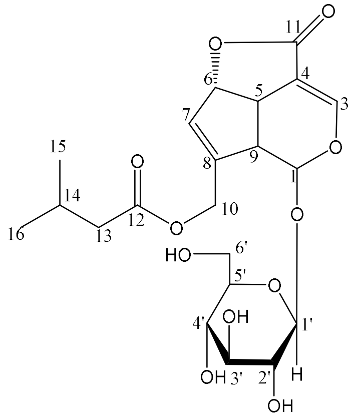

Elemental composition search on mass 479.15

m/z= 474.15-484.15

m/z	Theo. Mass	Delta (mmu)	RDB equiv.	Composition
479.1521	479.1524	-0.28	7.5	$C_{21}H_{28}O_{11}Na$

Figure S2. The ^{1}H NMR spectrum of **1** in CD_3OD, 500 MHz

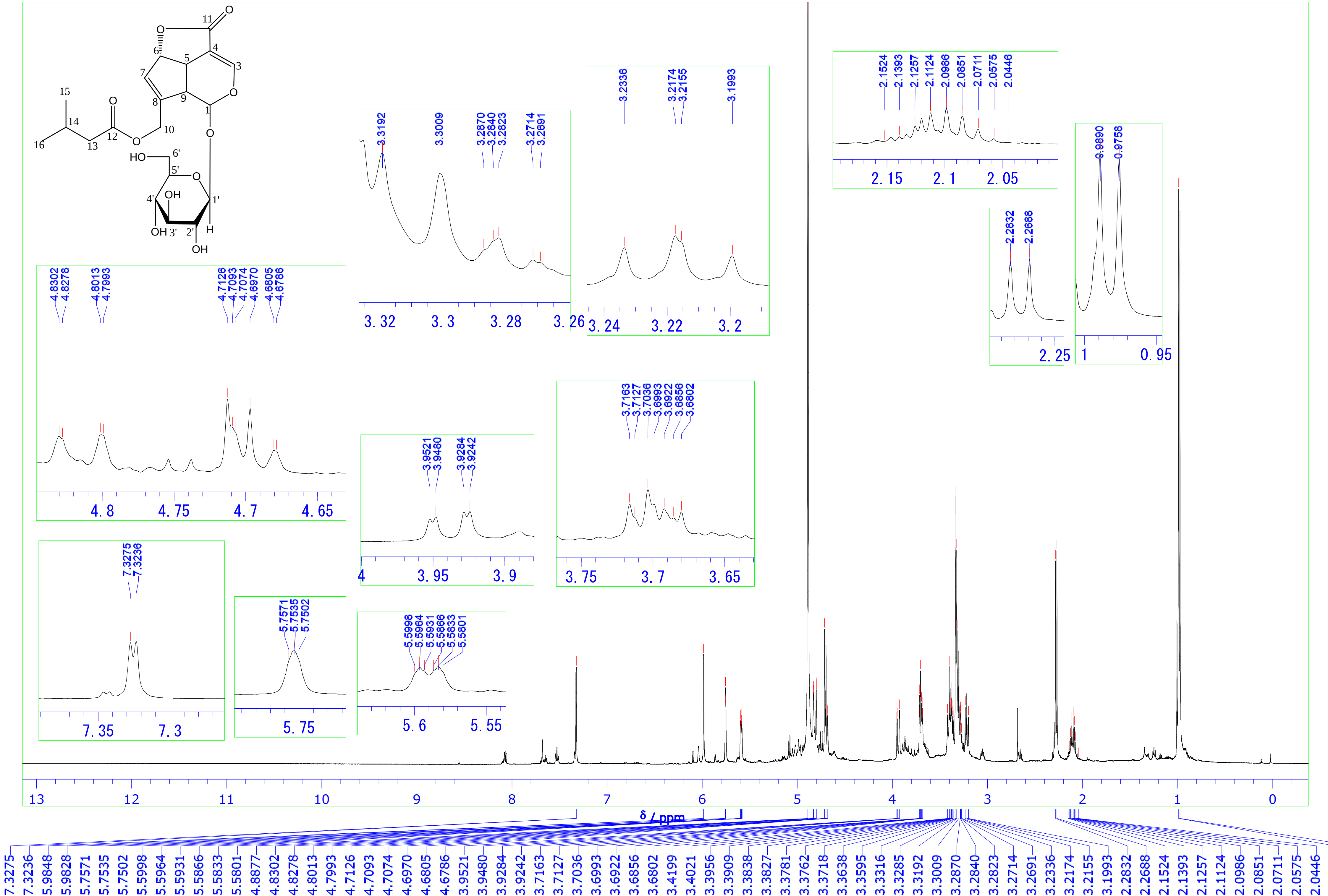

Figure S3. The ^{13}C NMR spectrum of **1** in CD_3OD, 175 MHz.

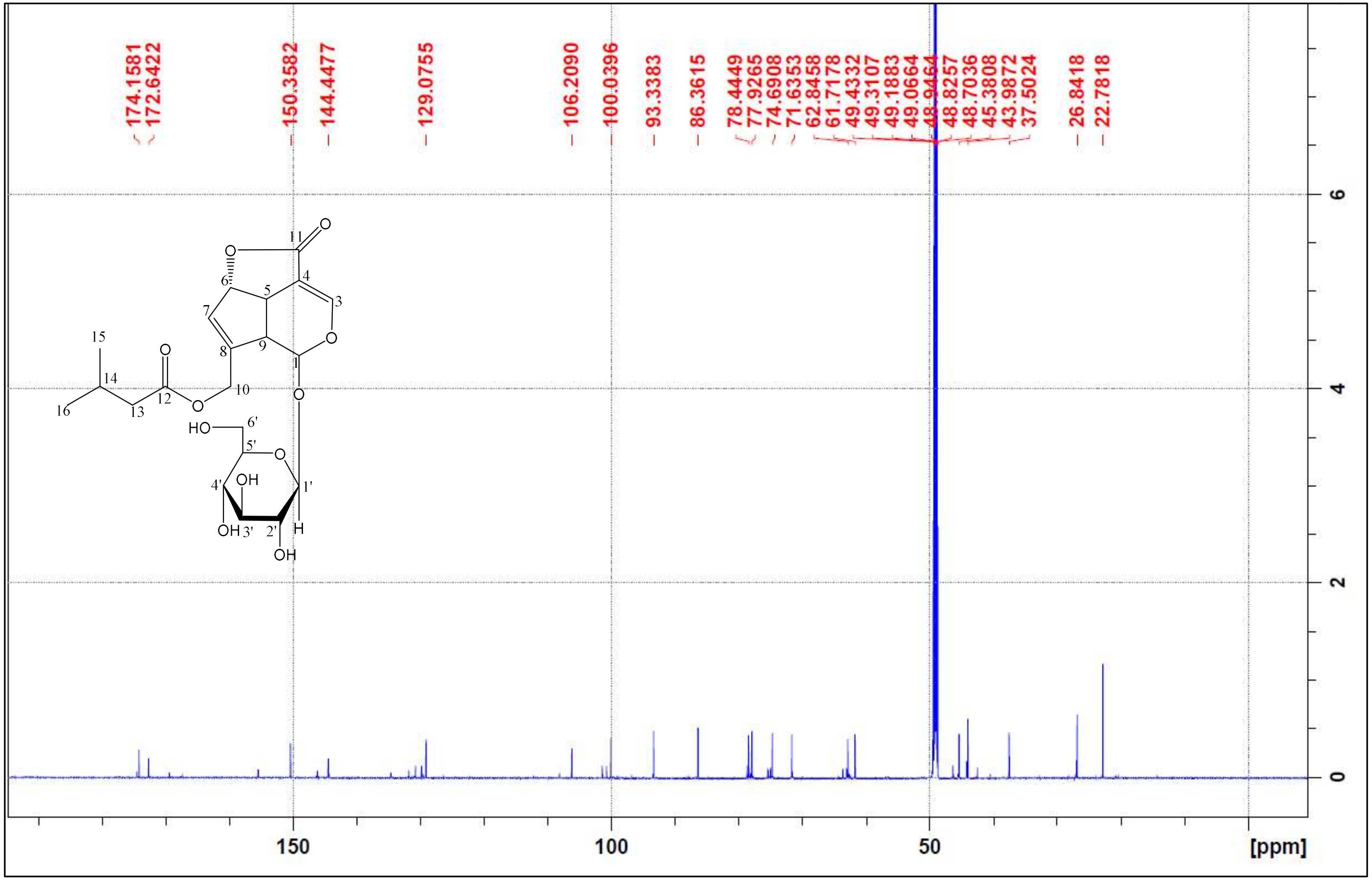

Figure S4. The DEPT-135 NMR spectrum of **1** in CD_3OD, 175 MHz.

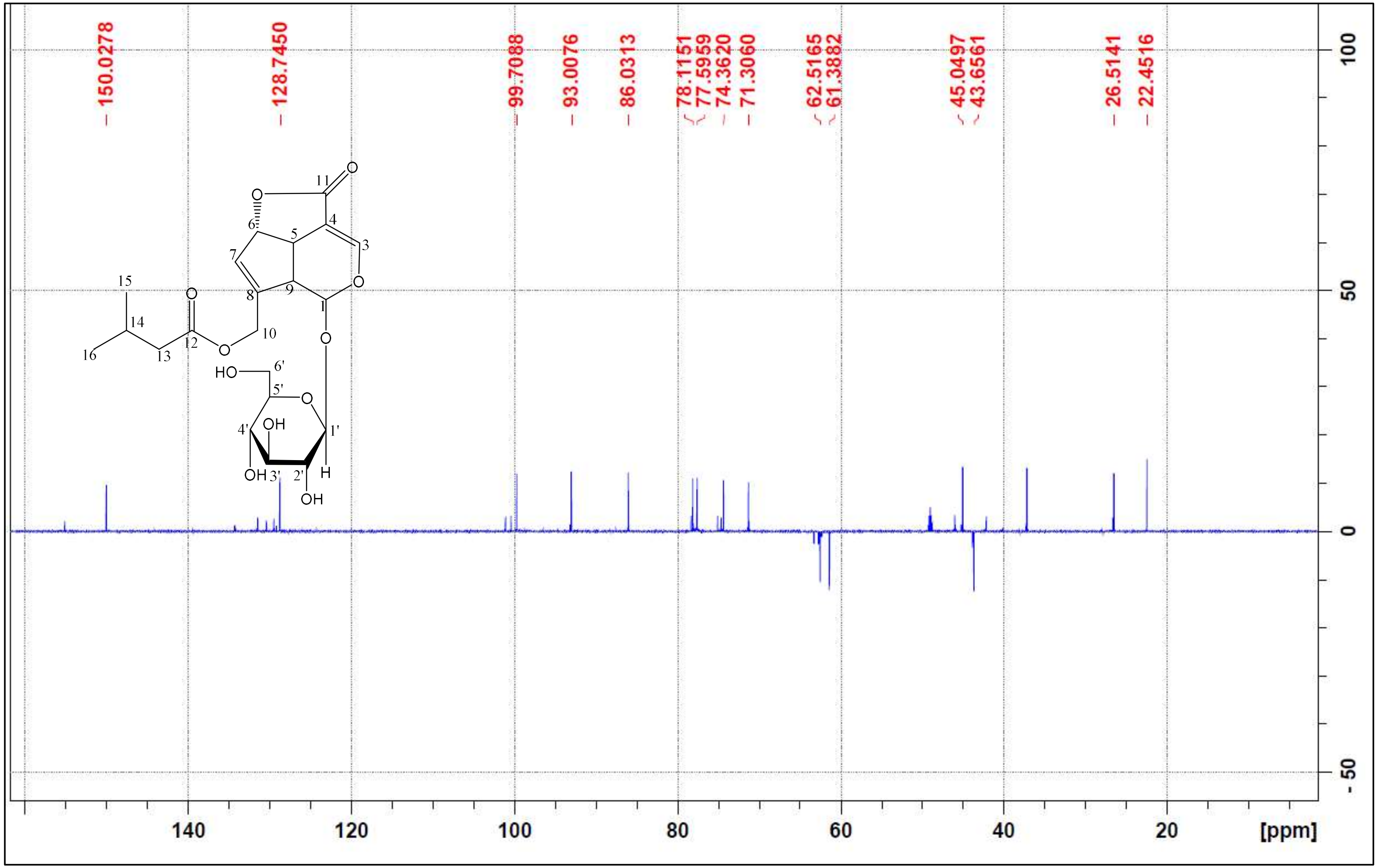

Figure S5. The COSY spectrum of **1** in CD_3OD, 500 MHz.

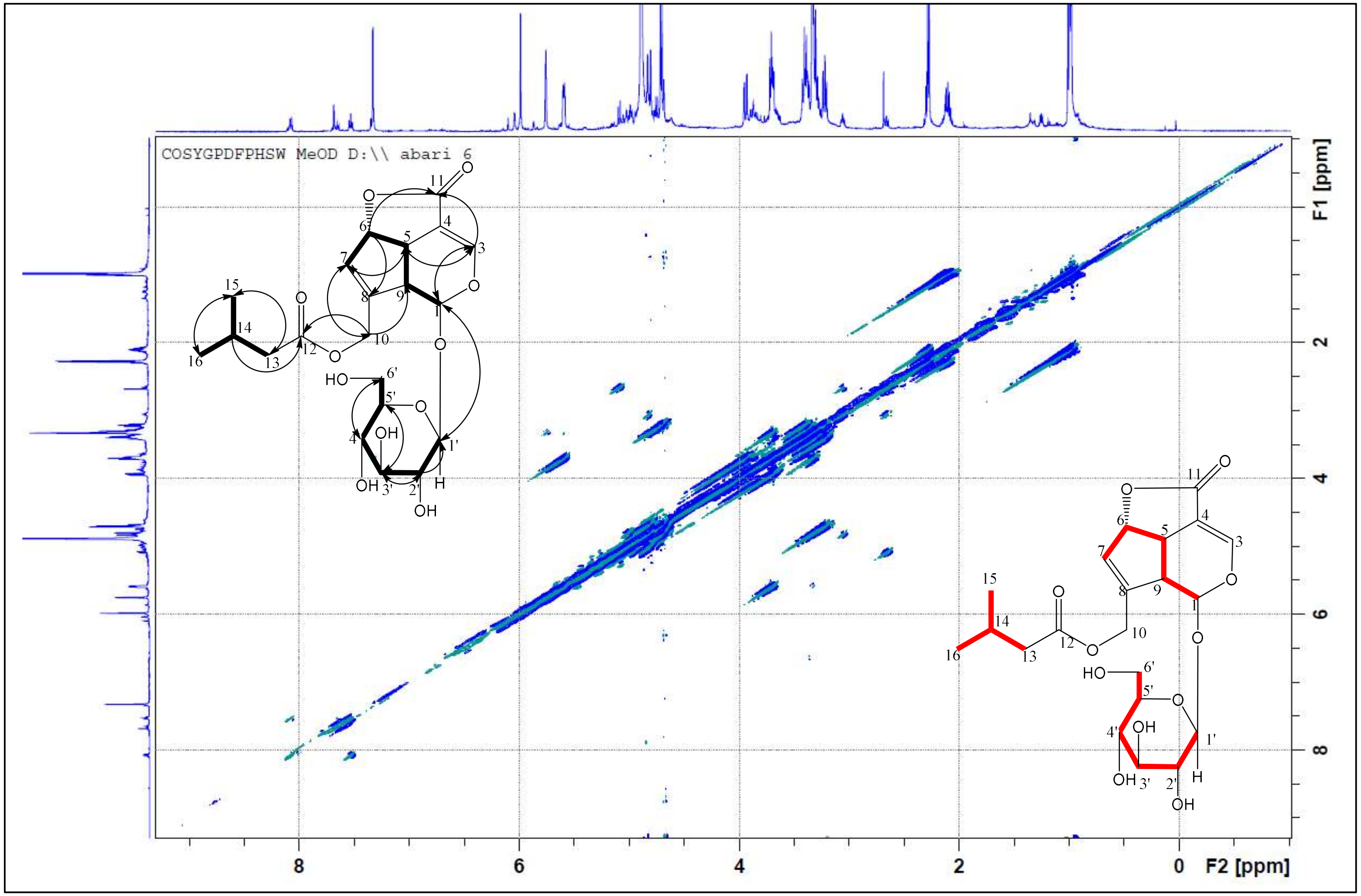

Figure S6. The HSQC spectrum of **1** in CD_3OD, 700 MHz

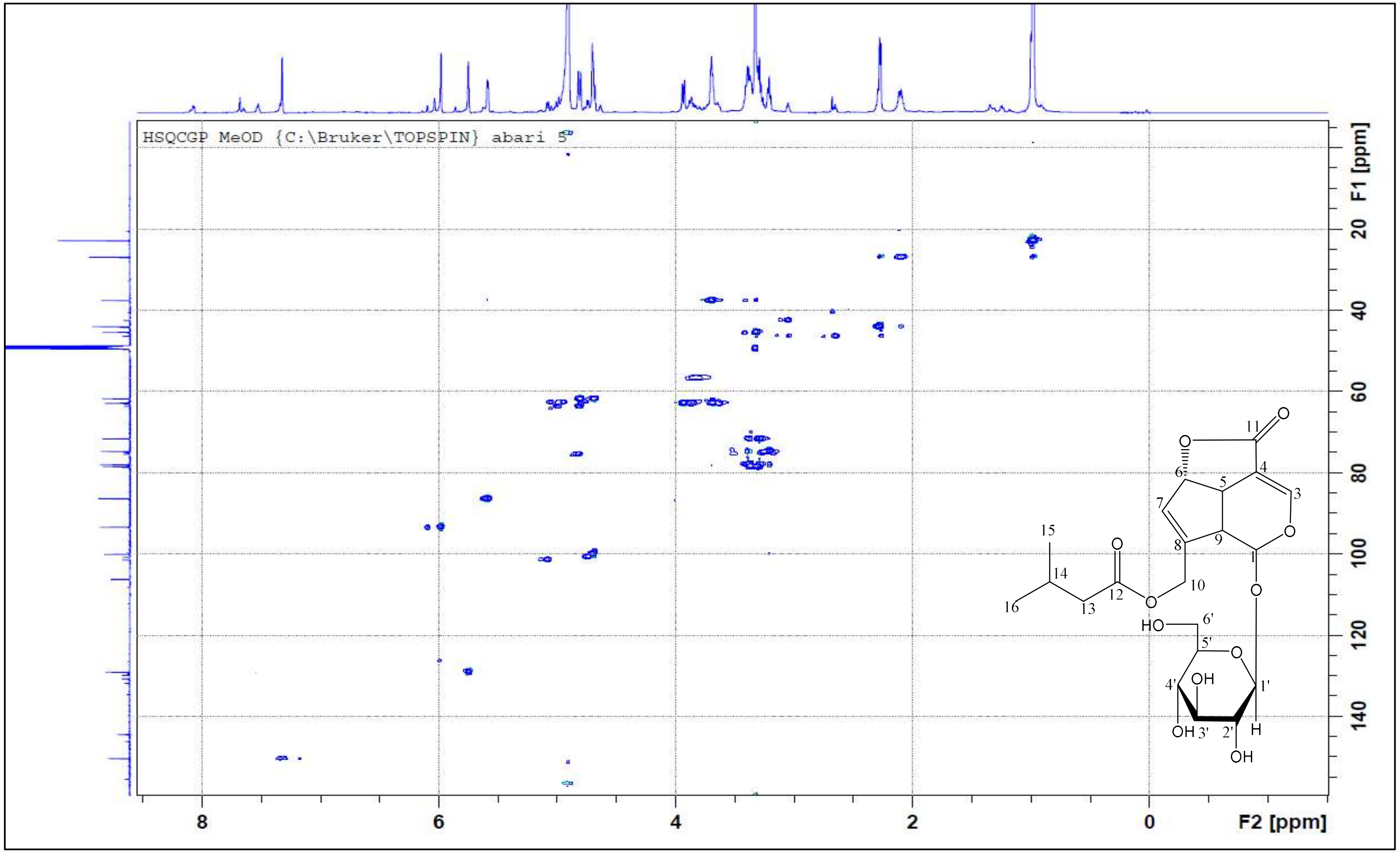

Figure S7. The HMBC spectrum of **1** in CD_3OD, 700 MHz.

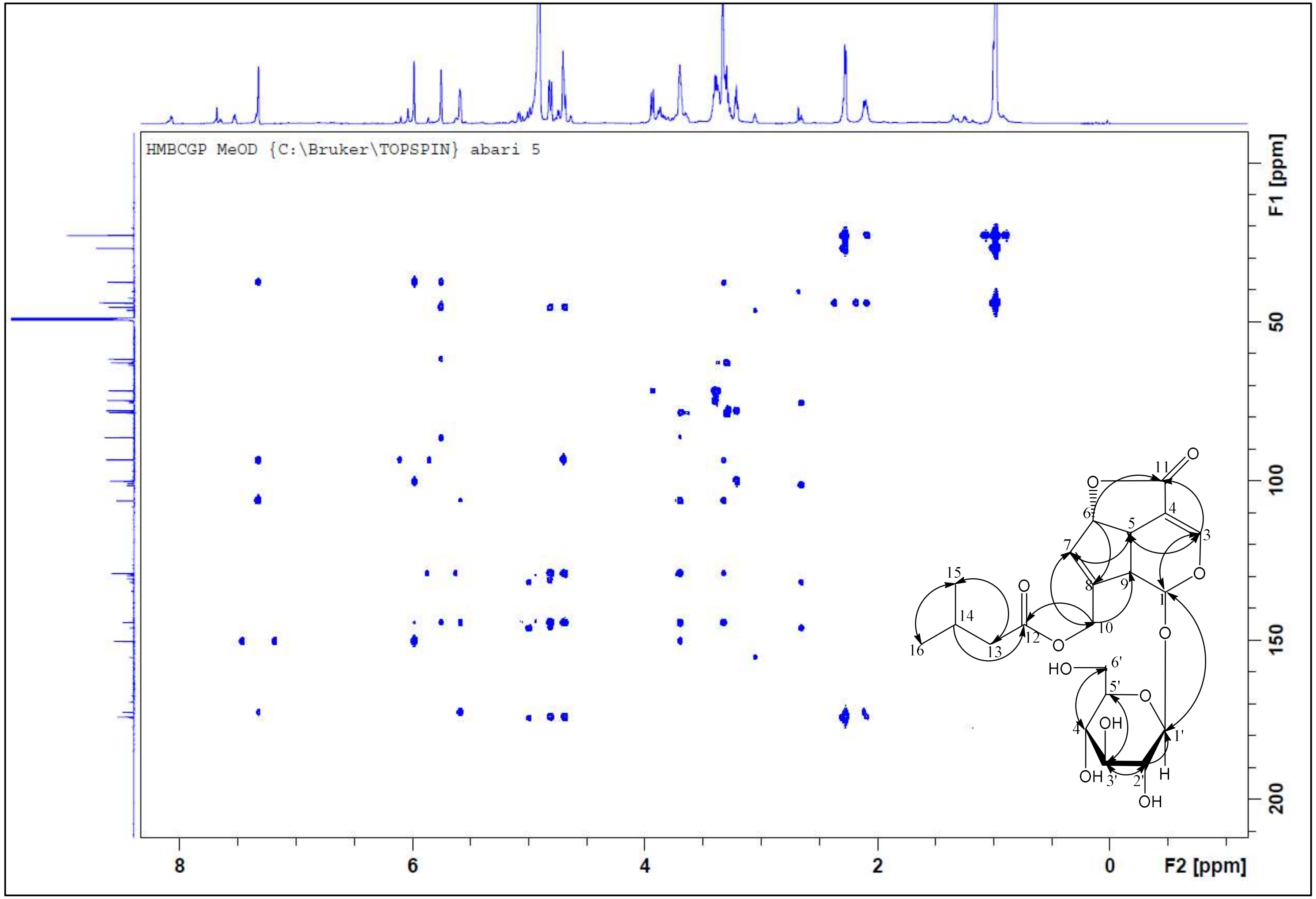

Figure S8. The NOESY spectrum of **1** in CD_3OD, 500 MHz.

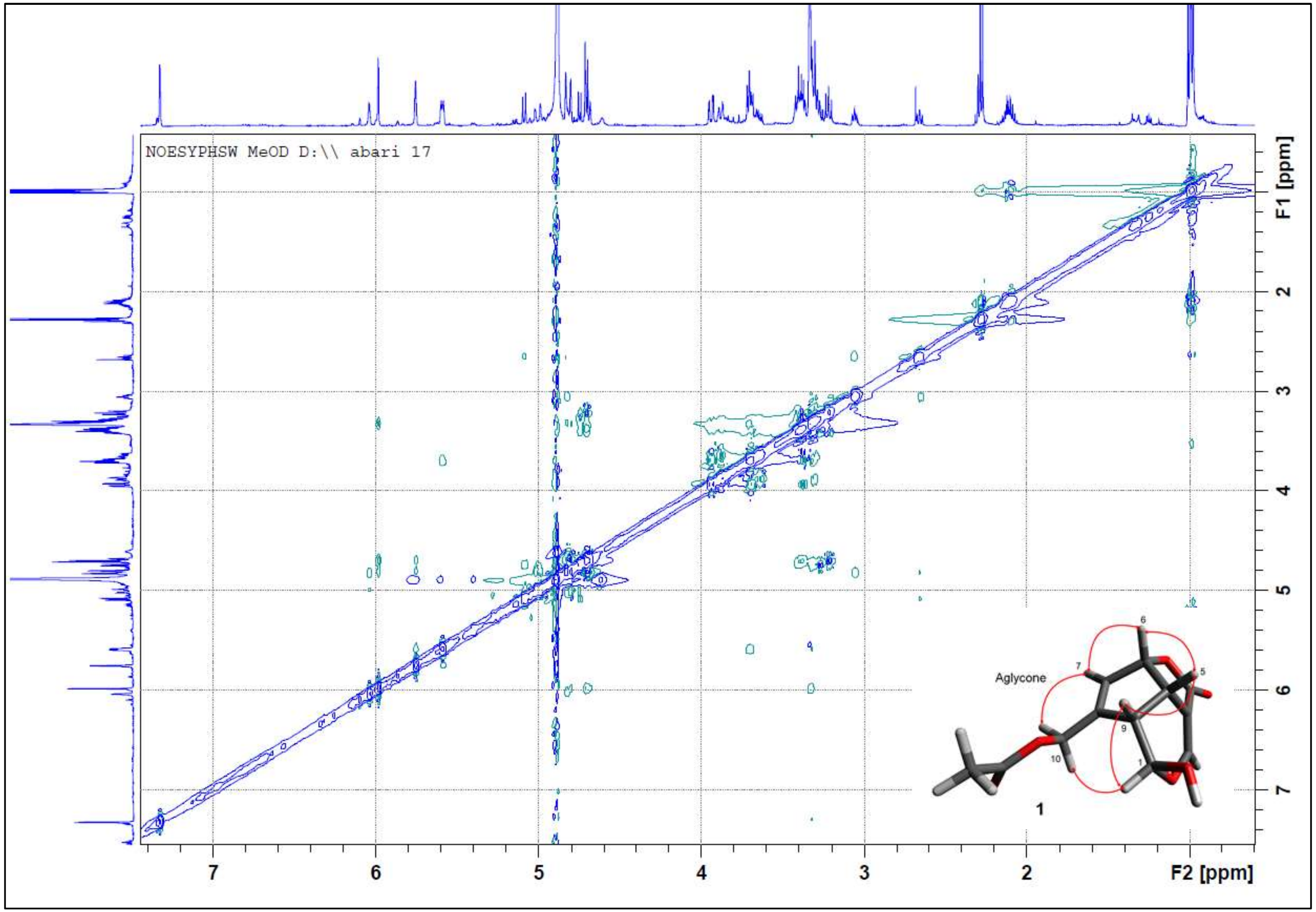

Figure S9. The UV spectrum of **1**.

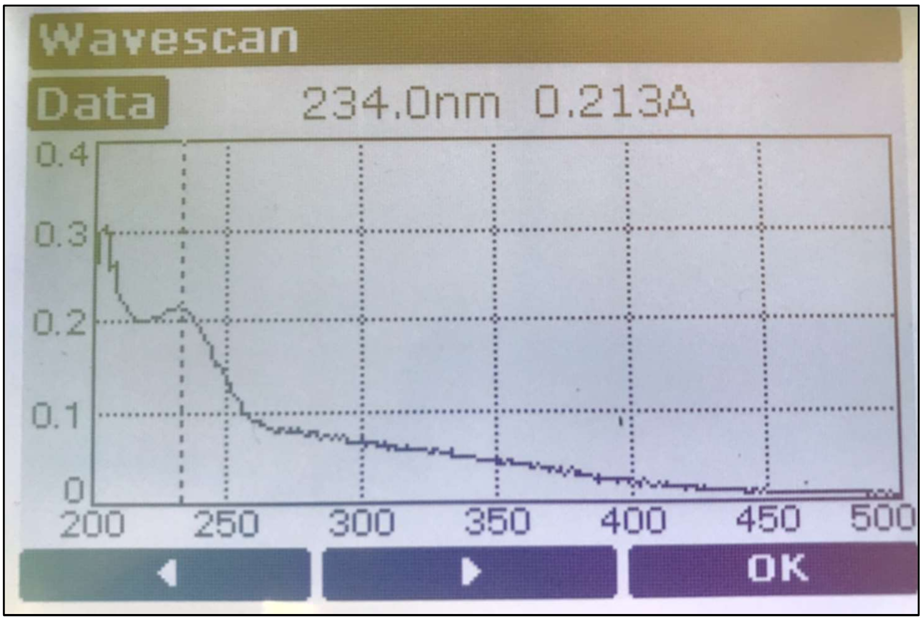

Figure S10. The IR spectrum of **1**.

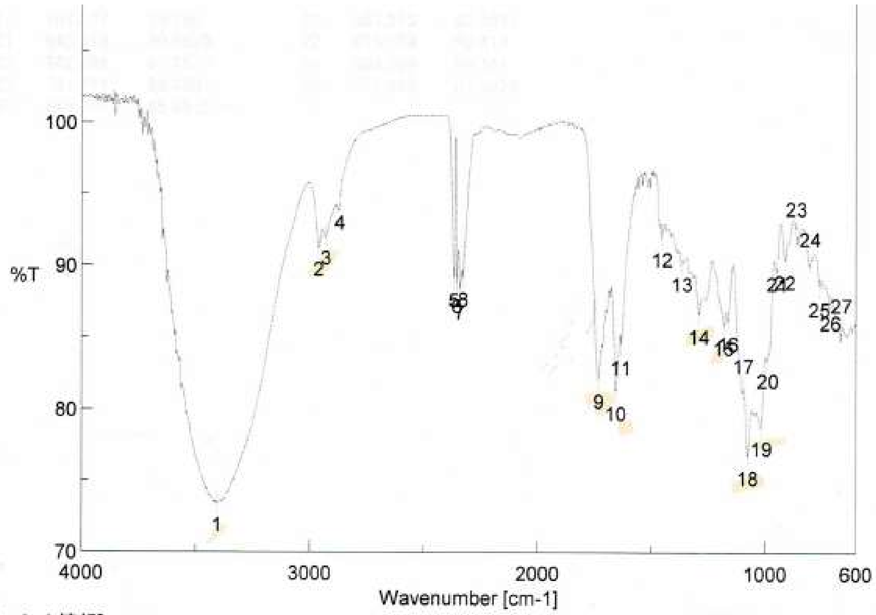

[コメント情報]

試料名	
コメント	
測定者	
所属	
会社	HU

[データ情報]

作成日時	2018/06/28 16:28
データタイプ	等間隔データ
横軸	Wavenumber [cm-1]
縦軸	%T
スタート	599.753 cm-1
エンド	7800.65 cm-1
データ間隔	0.964233 cm-1
データ数	7469

[測定情報]

機種名	FT/IR-4600typeA
シリアル番号	D015461786
測定日時	2018/06/28 16:28
光源	標準光源
検出器	TGS
積算回数	10
分解	4 cm-1
ゼロフィリング	On
アポダイゼーション	Cosine
ゲイン	Auto (2)
アパーチャー	Auto (7.1 mm)
スキャンスピード	Auto (2 mm/sec)
フィルタ	Auto (30000 Hz)

[ピーク検出結果]

No.	位置	強度	No.	位置	強度
1	3405.67	73.4066	2	2960.2	91.2685
3	2928.38	92.0789	4	2867.63	94.546
5	2367.19	89.1117	6	2353.69	88.6924
7	2341.16	88.2627	8	2326.7	89.1665
9	1732.73	82.1065	10	1658.48	81.2529

[ピーク検出結果]

No.	位置	強度	No.	位置	強度
15	1183.11	85.8152	16	1163.83	86.0948
17	1096.33	81.4734	18	1077.05	76.7278
19	1017.27	78.795	20	987.375	83.5641
21	945.913	90.2839	22	913.129	90.413
23	862.989	92.4537	24	803.206	90.341
25	761.744	88.4887	26	712.569	87.5625
27	664.357	85.6575			

Figure S11. The HRESIMS spectrum of **2**.

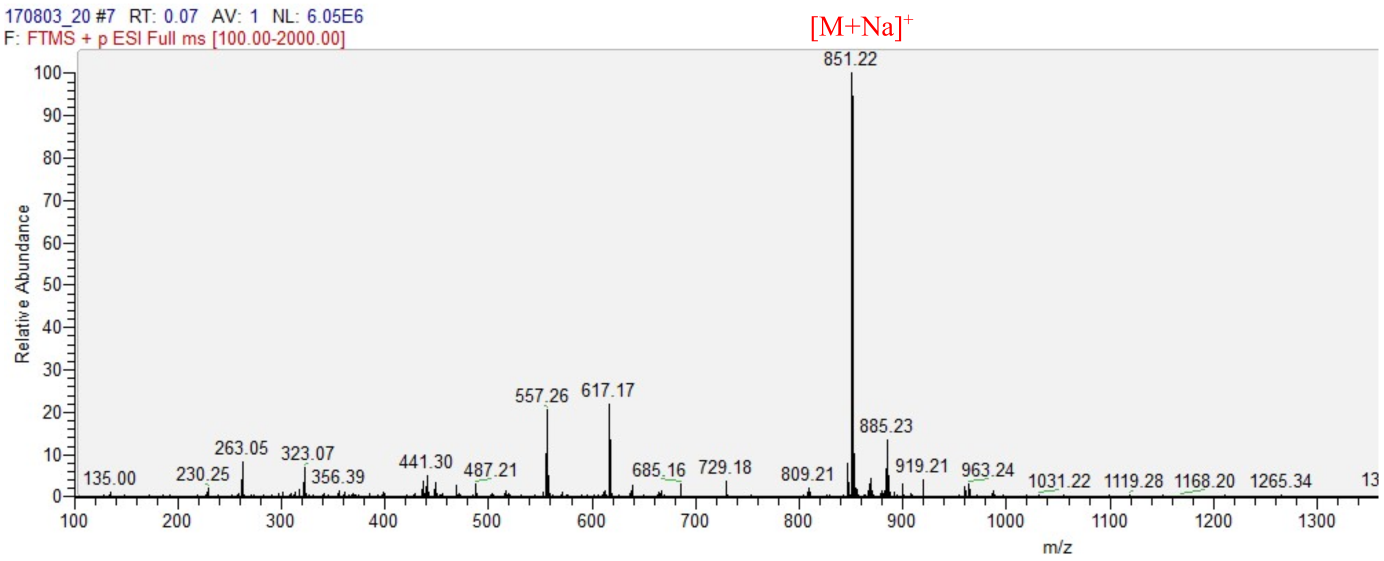

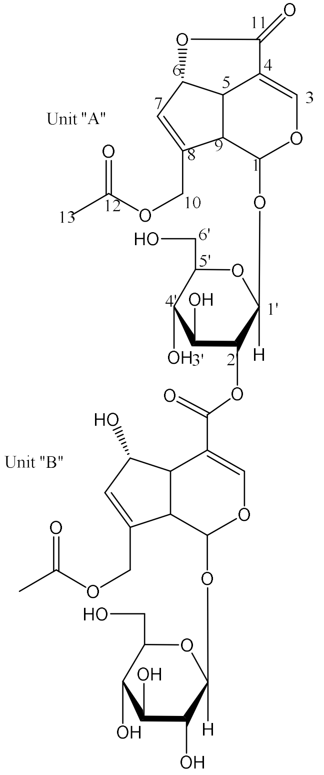

Elemental composition search on mass 851.22

m/z= 846.22-856.22

m/z	Theo. Mass	Delta (mmu)	RDB equiv.	Composition
851.2214	851.2216	-0.27	14.5	$C_{36}H_{44}O_{22}Na$

Figure S12. The ^{1}H NMR spectrum of **2** in CD_3OD, 500 MHz

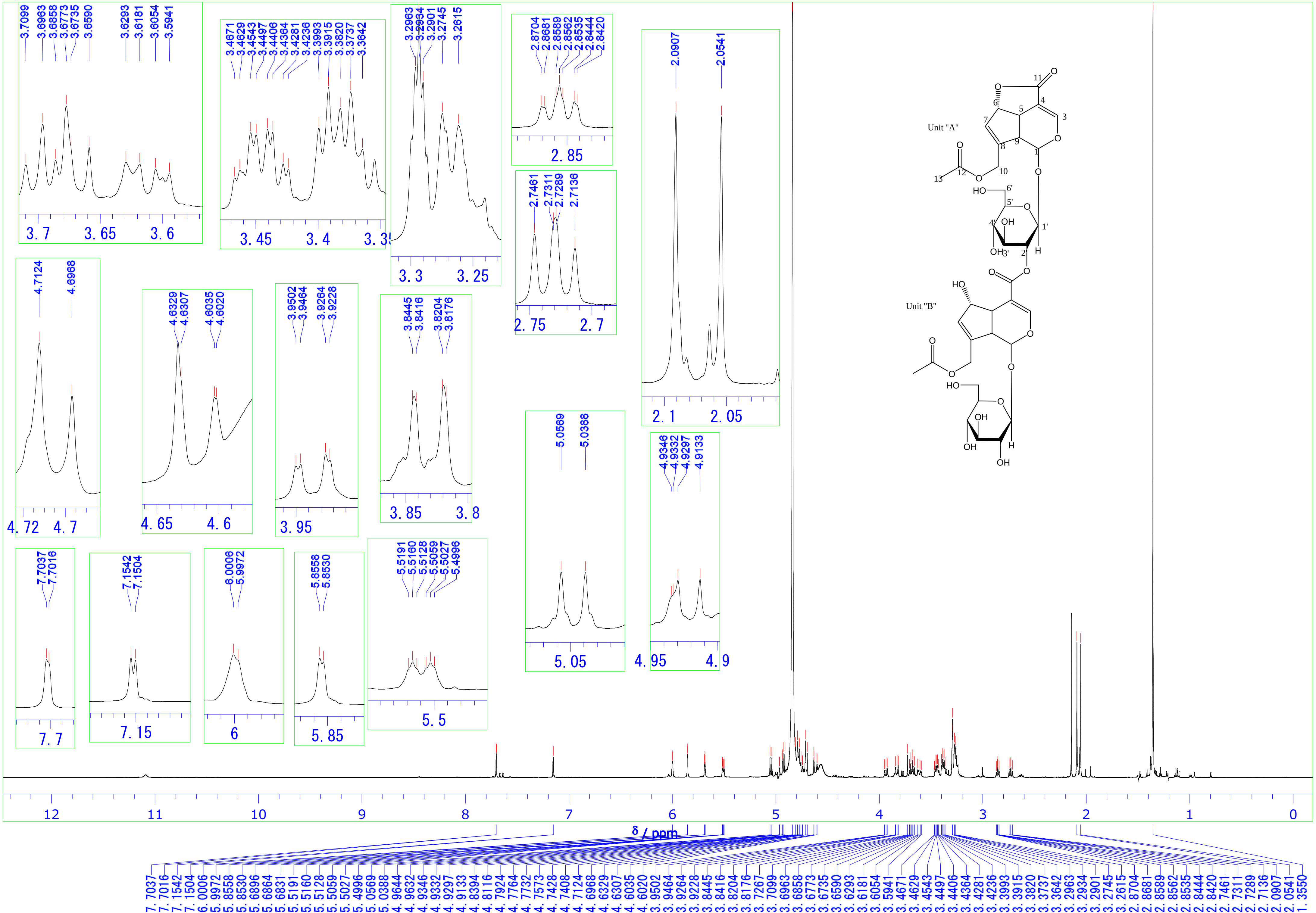

Figure S13. The ^{13}C NMR spectrum of **2** in CD_3OD, 125 MHz.

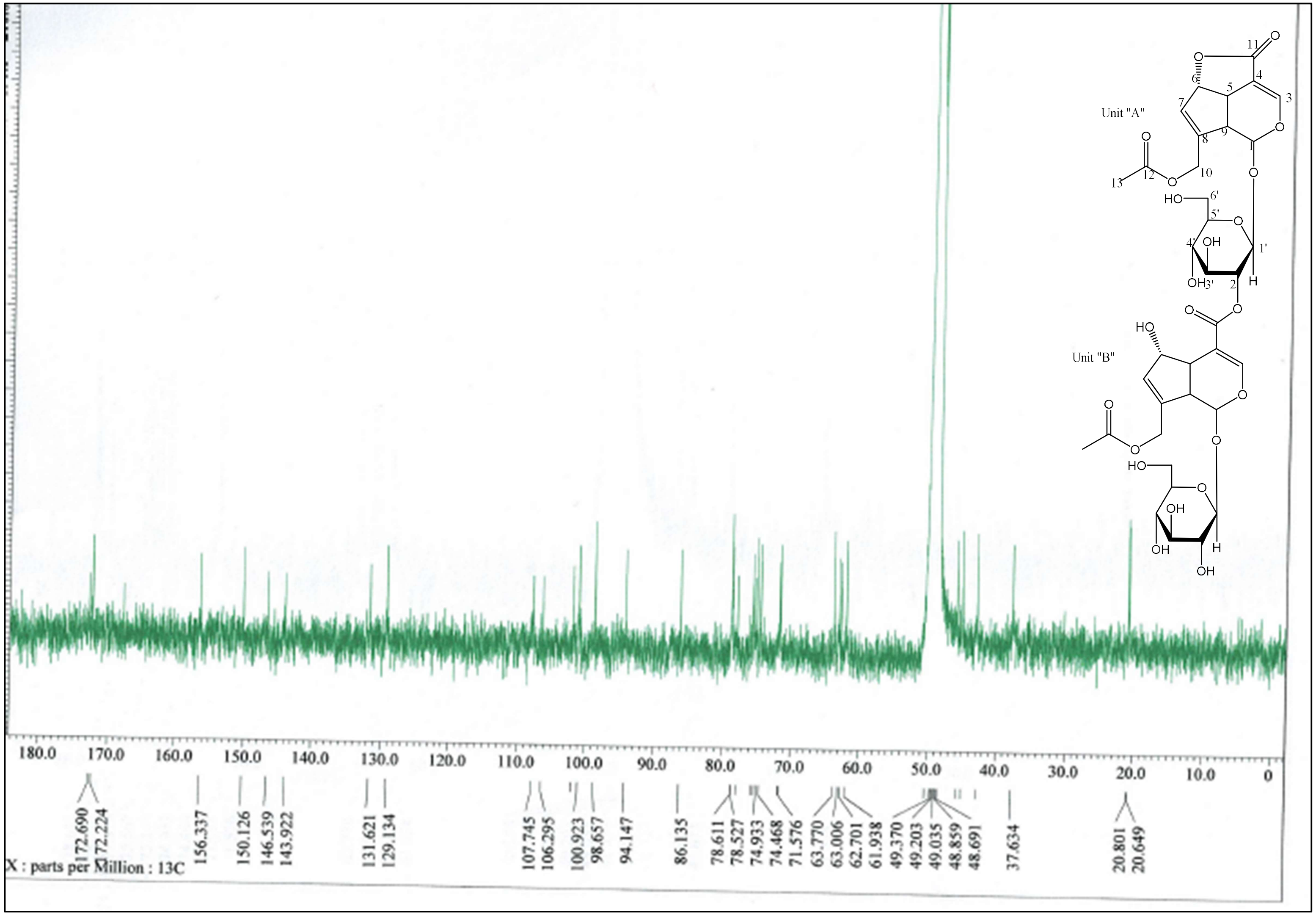

Figure S14. The ^{13}C NMR spectrum of **2** in CD_3OD, 125 MHz (magnified)

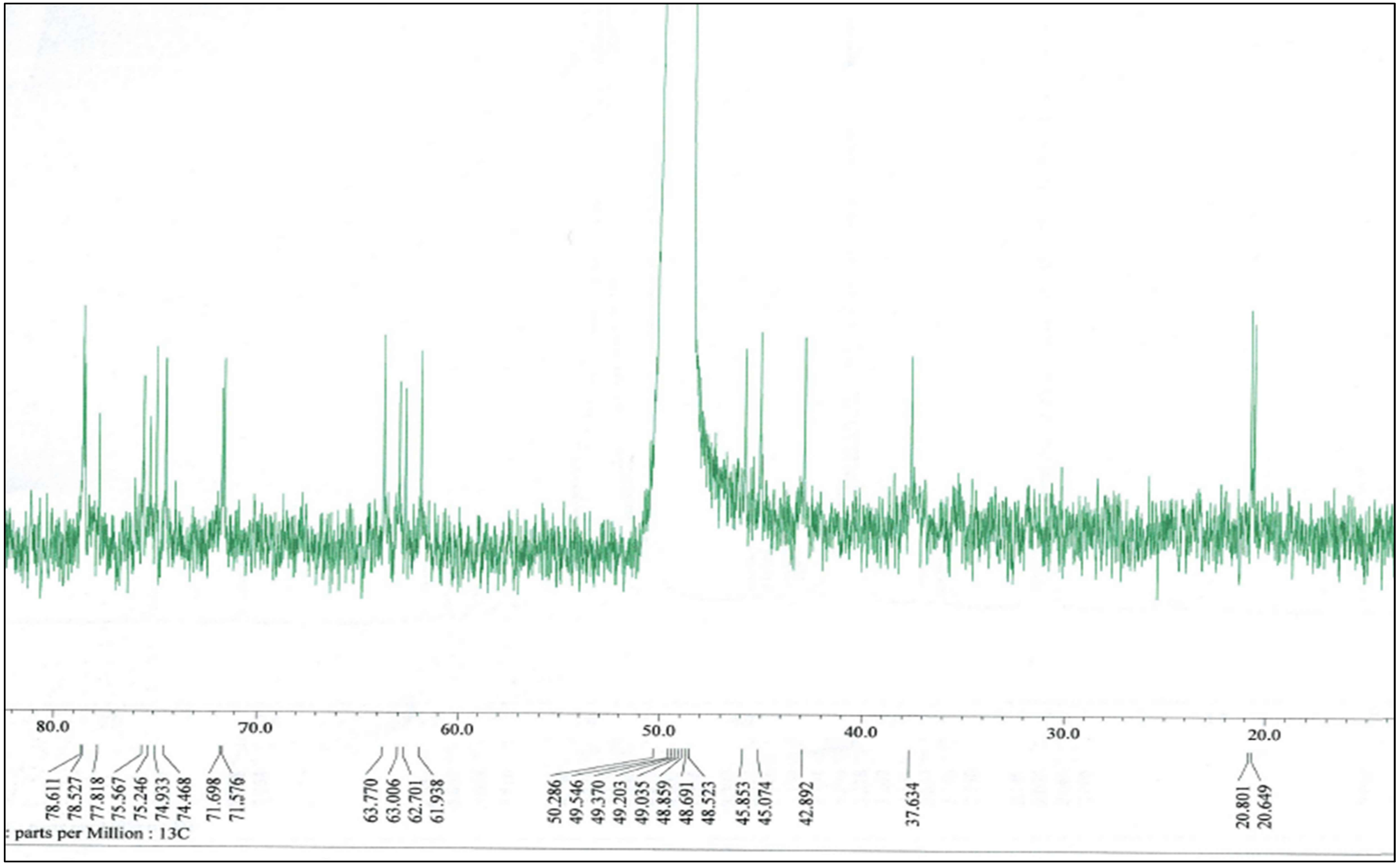

Figure S15. The DEPT-135 NMR spectrum of **2** in CD_3OD, 175 MHz

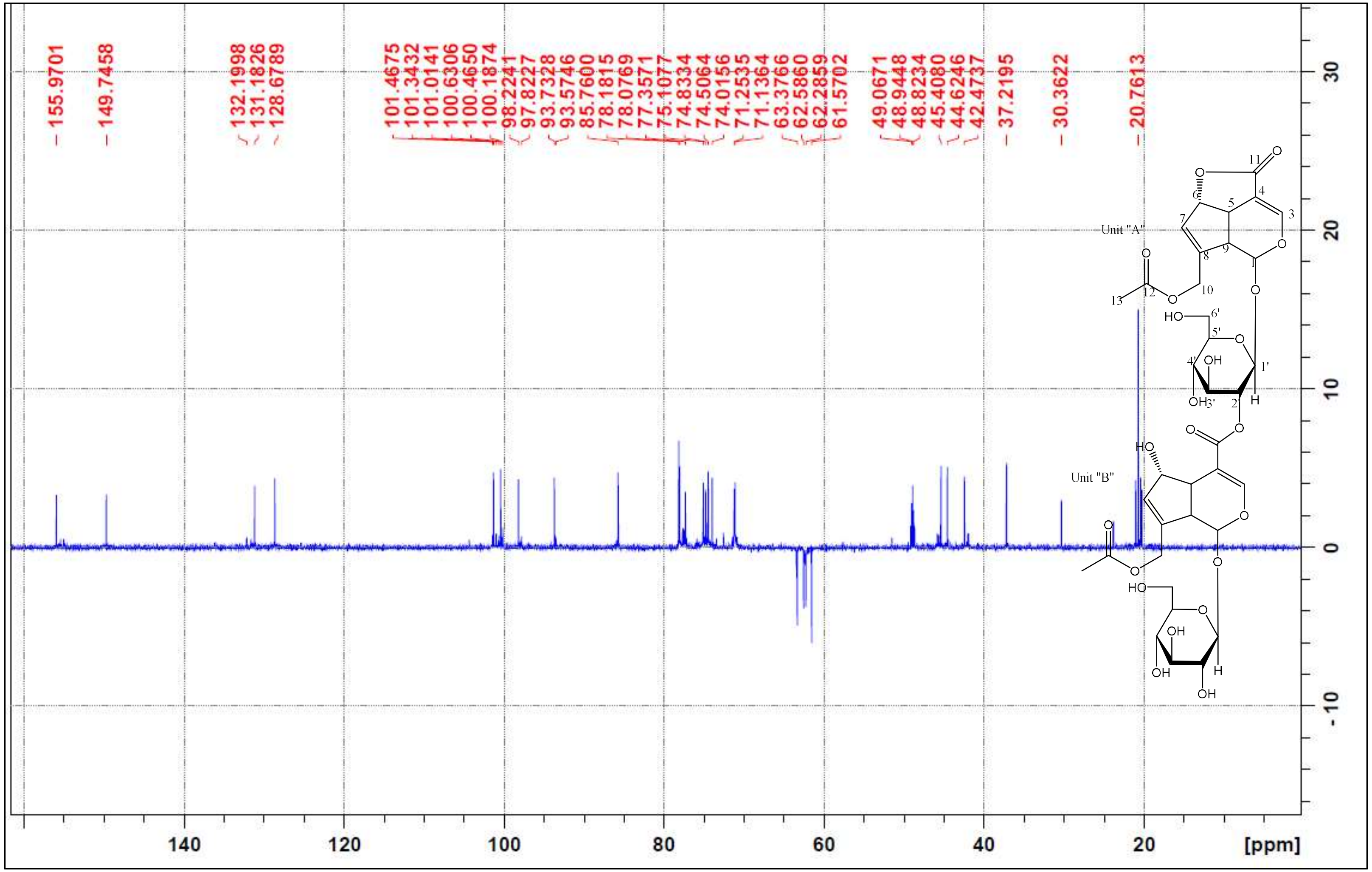

Figure S16. The COSY spectrum of **2** in CD_3OD, 500 MHz.

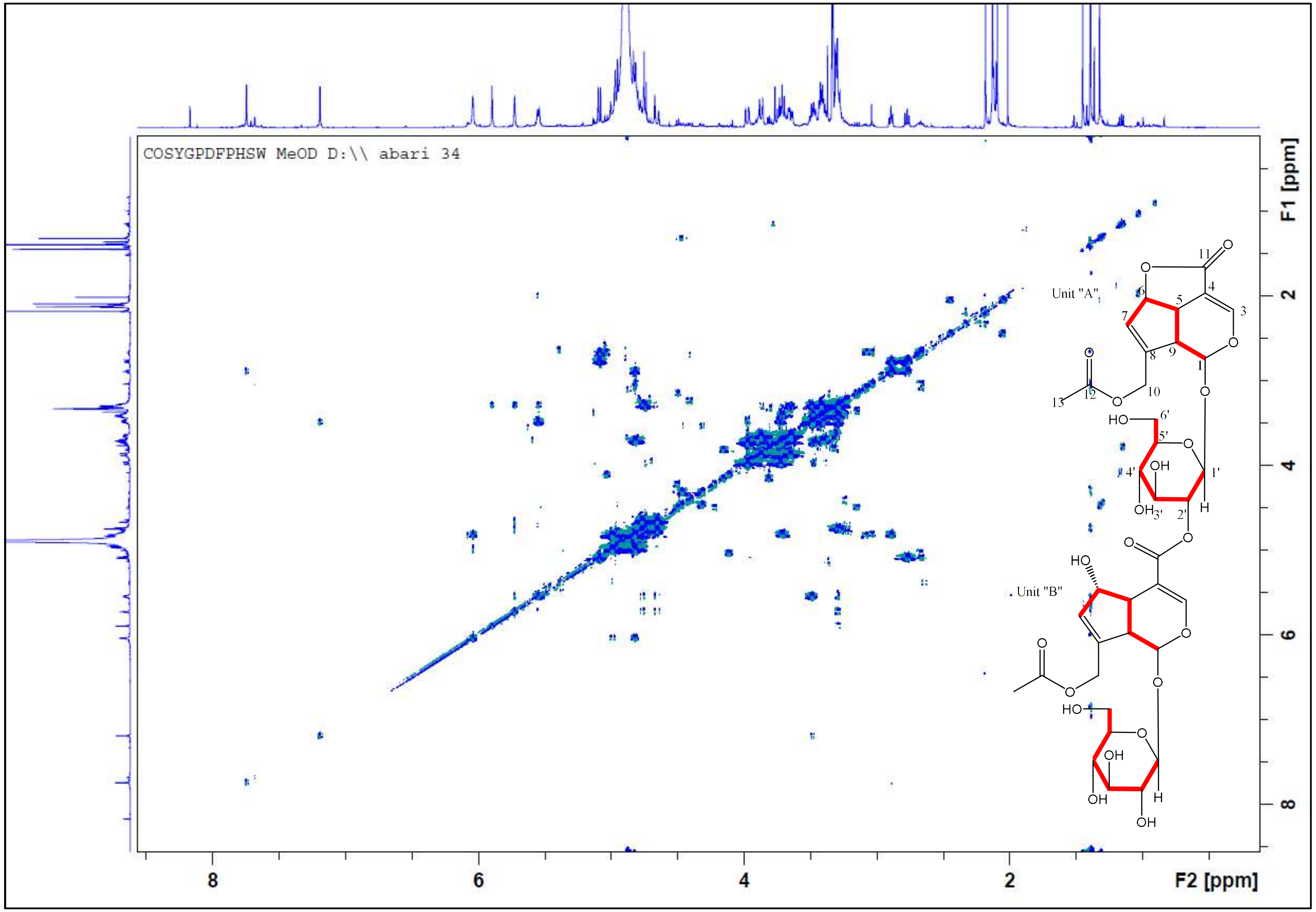

Figure S17. The HSQC spectrum of **2** in CD_3OD, 700 MHz

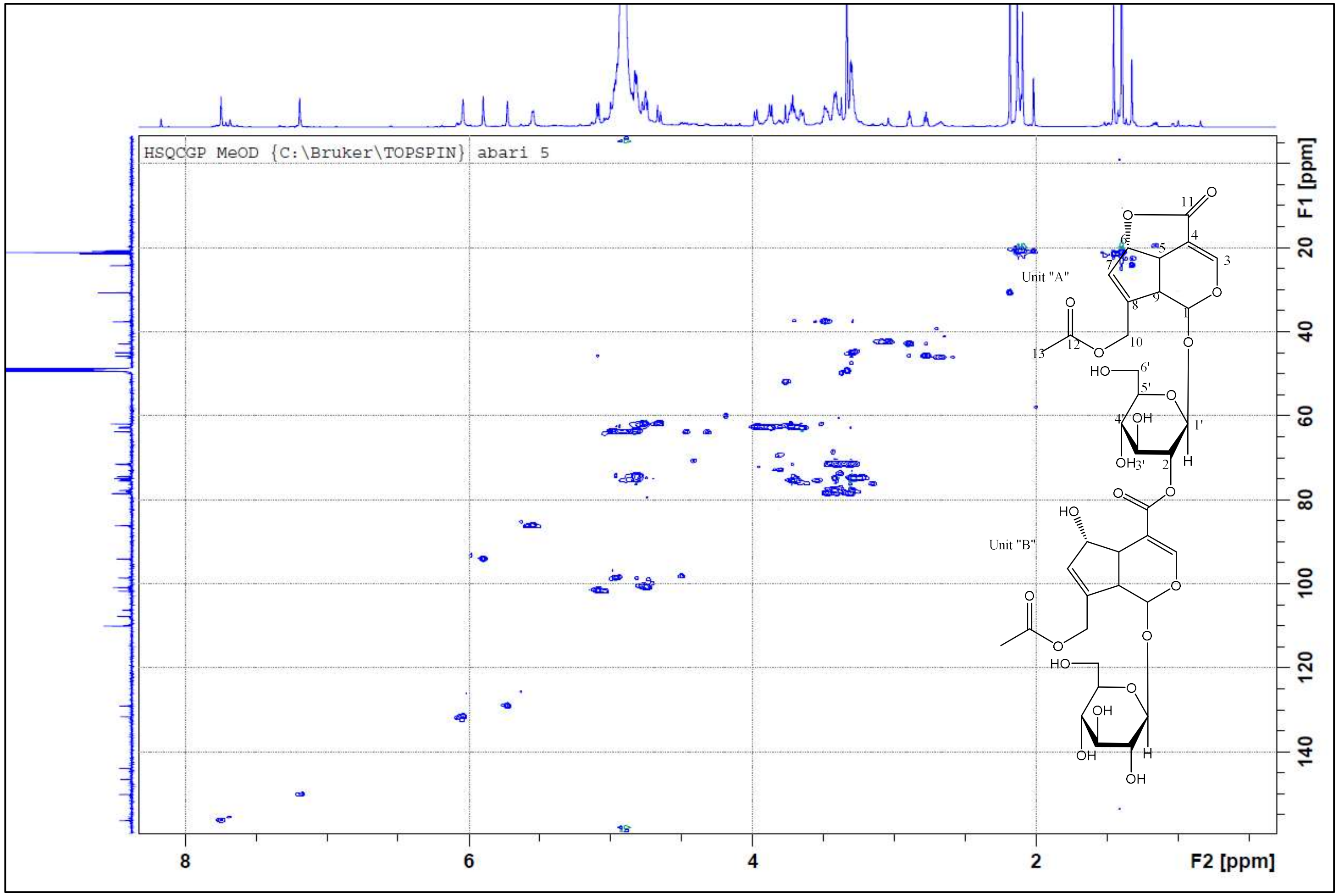

Figure S18. The HMBC spectrum of **2** in CD_3OD, 700 MHz.

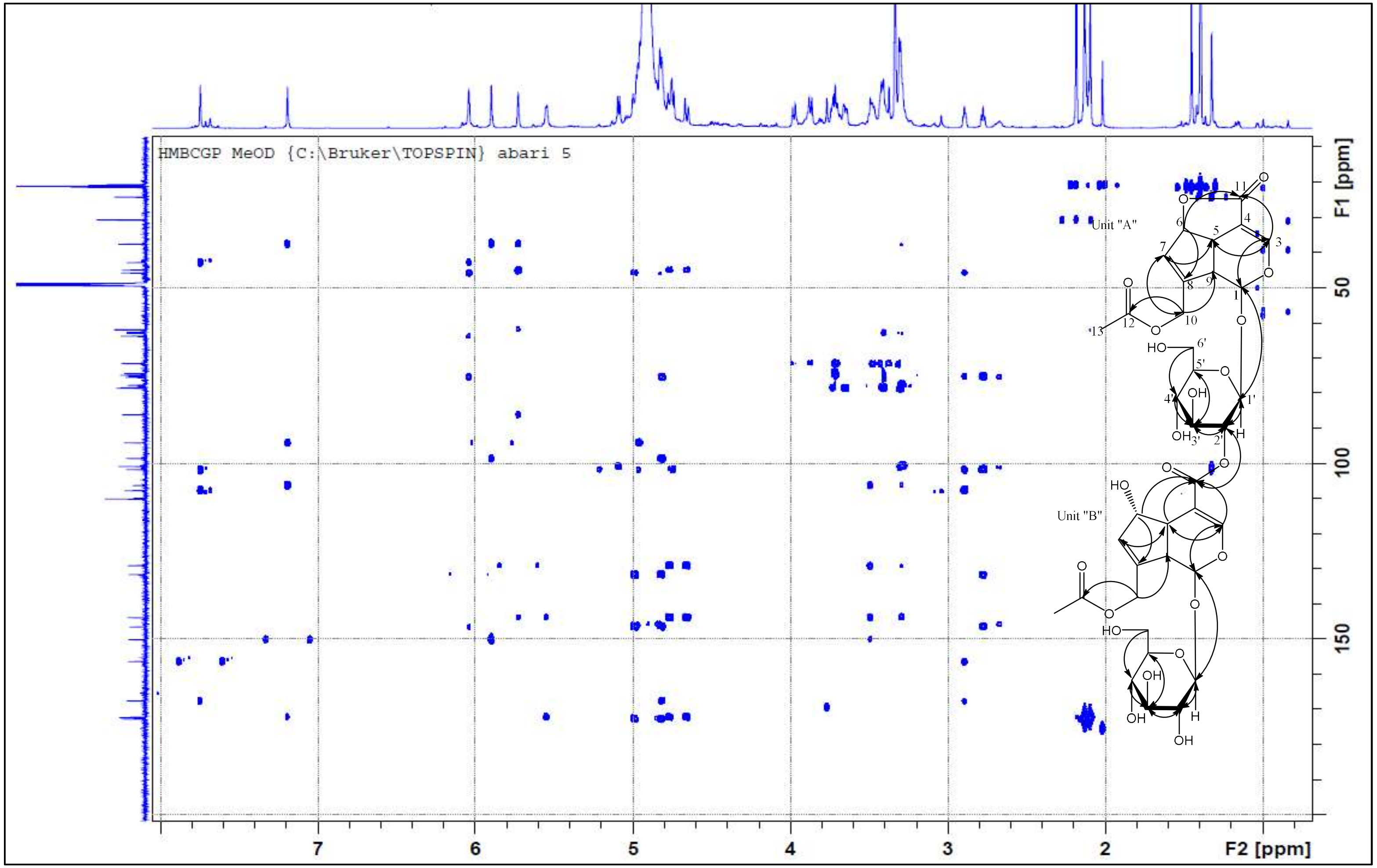

Figure S19. The NOESY spectrum of **2** in CD_3OD, 500 MHz.

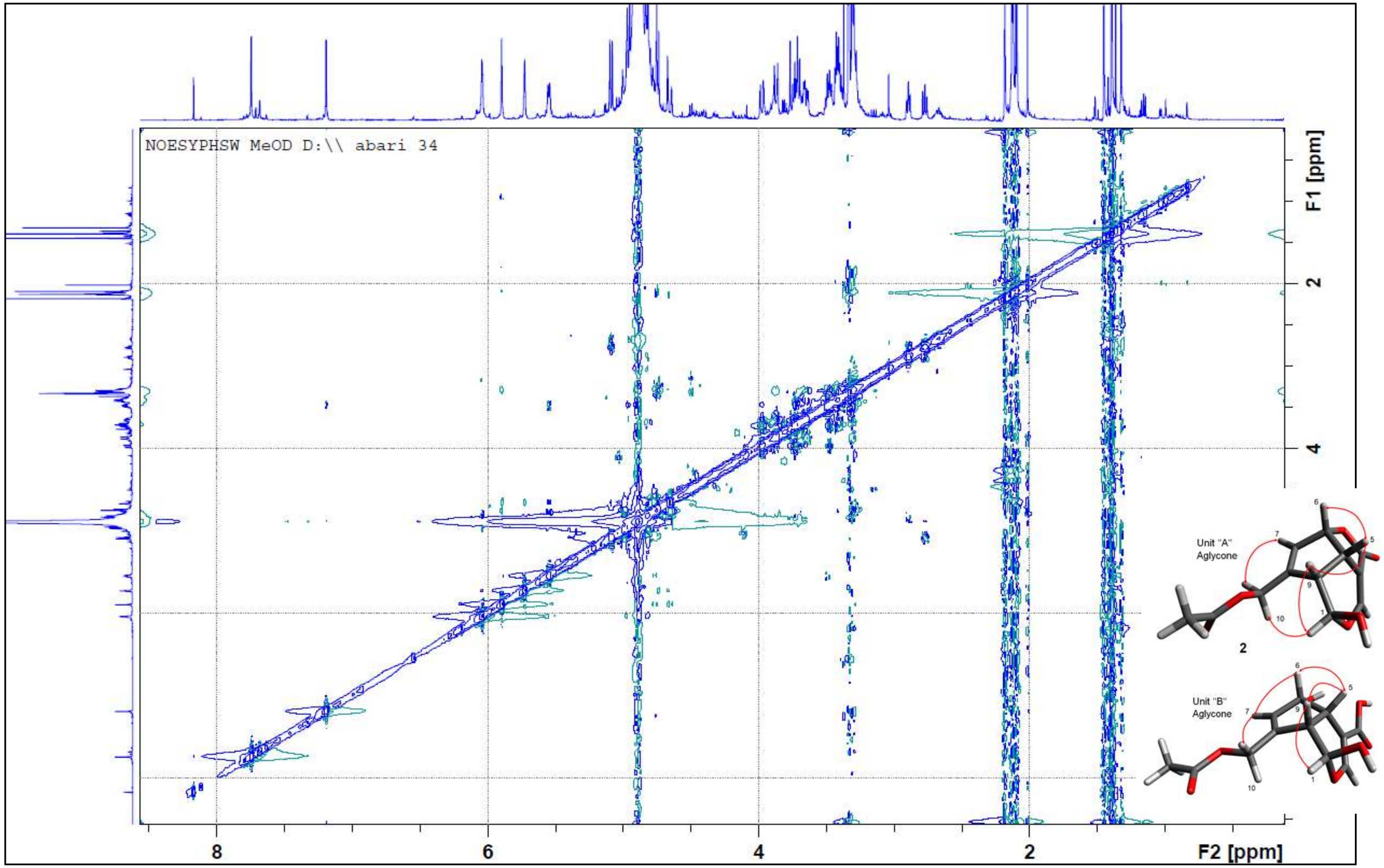

Figure S20. The UV spectrum of **2**

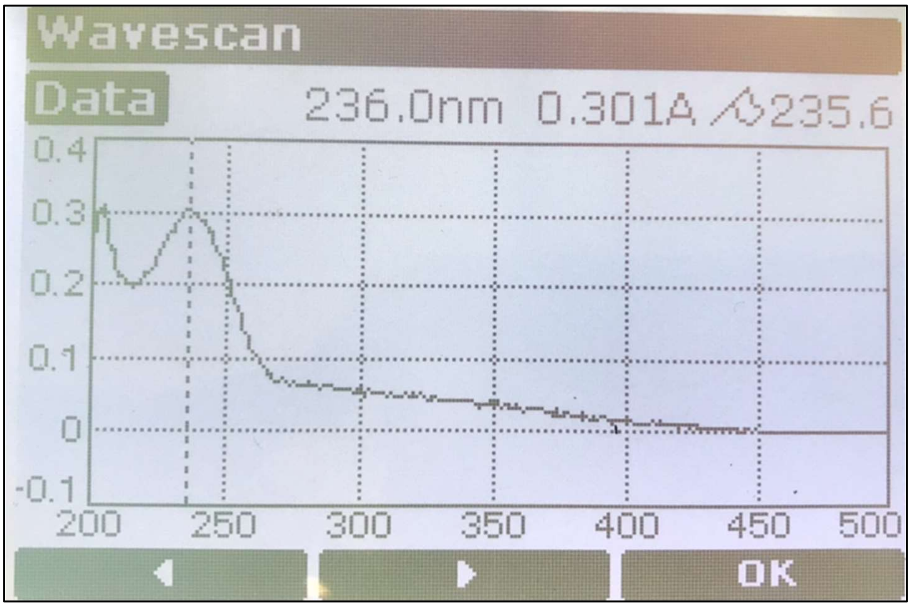

Figure S21. The IR spectrum of **2**.

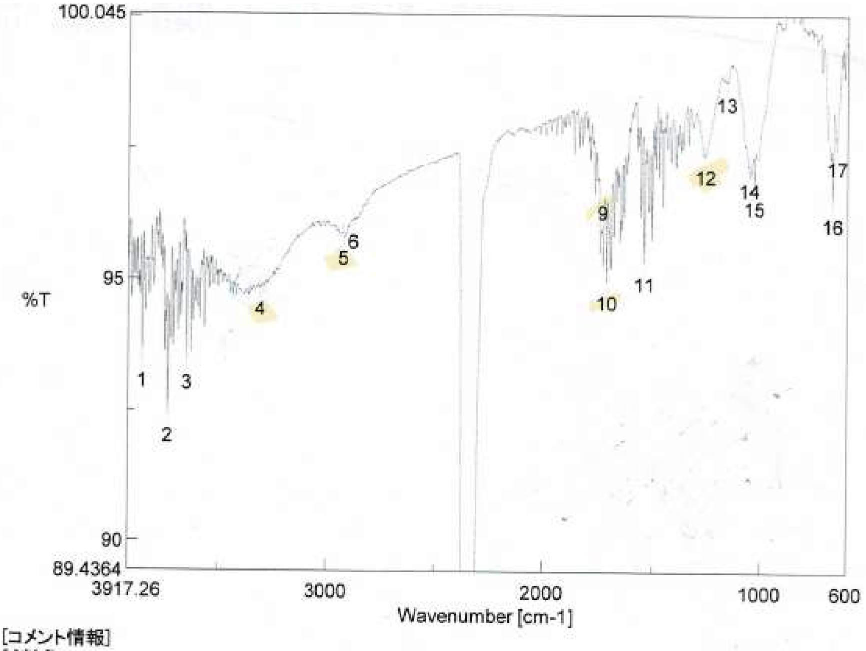

[コメント情報]

試料名	
コメント	
測定者	
所属	
会社	HU

[データ情報]

作成日時	2018/06/28 16:42
更新日時	2018/07/04 16:29
データタイプ	等間隔データ
横軸	Wavenumber [cm-1]
縦軸	%T
スタート	599.753 cm-1
エンド	7800.65 cm-1
データ間隔	0.964233 cm-1
データ数	7469

[測定情報]

機種名	FT/IR-4600typeA
シリアル番号	D015461786
測定日時	2018/06/28 16:42
光源	標準光源
検出器	TGS
積算回数	10
分解	4 cm-1
ゼロフィリング	On
アポダイゼーション	Cosine
ゲイン	Auto (2)
アパーチャー	Auto (7.1 mm)
スキャンスピード	Auto (2 mm/sec)
フィルタ	Auto (30000 Hz)

[ピーク検出結果]

No.	位置	強度	No.	位置	強度
1	3852.11	93.466	2	3733.51	92.4263
3	3647.7	93.4297	4	3309.25	94.8277
5	2924.52	95.8138	6	2879.2	96.1213
7	2364.3	80.1815	8	2341.16	81.3431
9	1735.62	95.8677	10	1716.34	94.9707
11	1540.85	95.3439	12	1260.25	97.3865
13	1161.9	98.7761	14	1056.8	97.1332

[ピーク検出結果]

No.	位置	強度	No.	位置	強度
15	1032.69	96.7891	16	669.178	96.4733
17	648.929	97.5611			

Figure S22. The HRESIMS spectrum of **3**.

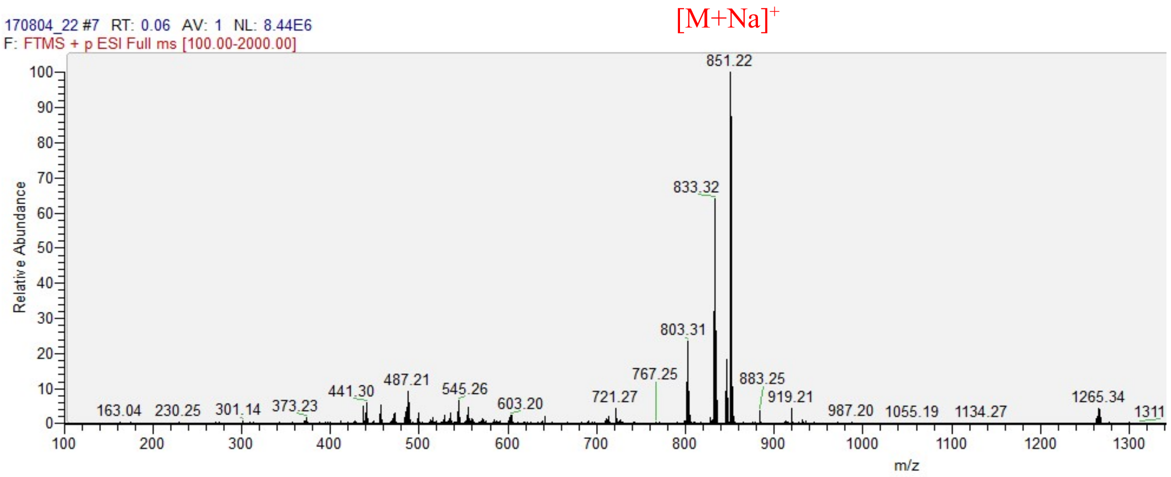

Elemental composition search on mass 851.22

m/z= 846.22-856.22

m/z	Theo. Mass	Delta (mmu)	RDB equiv.	Composition
851.2212	851.2216	-0.45	14.5	$C_{36}H_{44}O_{22}Na$

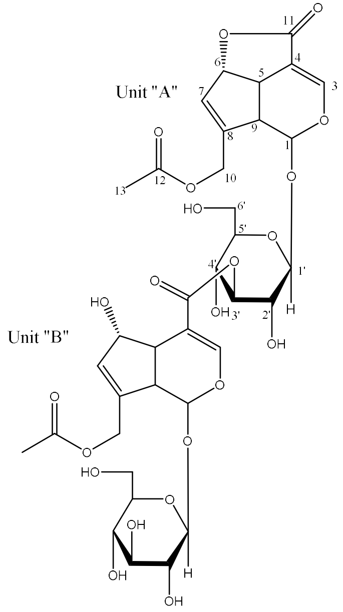

Figure S23. The ^{1}H NMR spectrum of **3** in CD_3OD, 500 MHz.

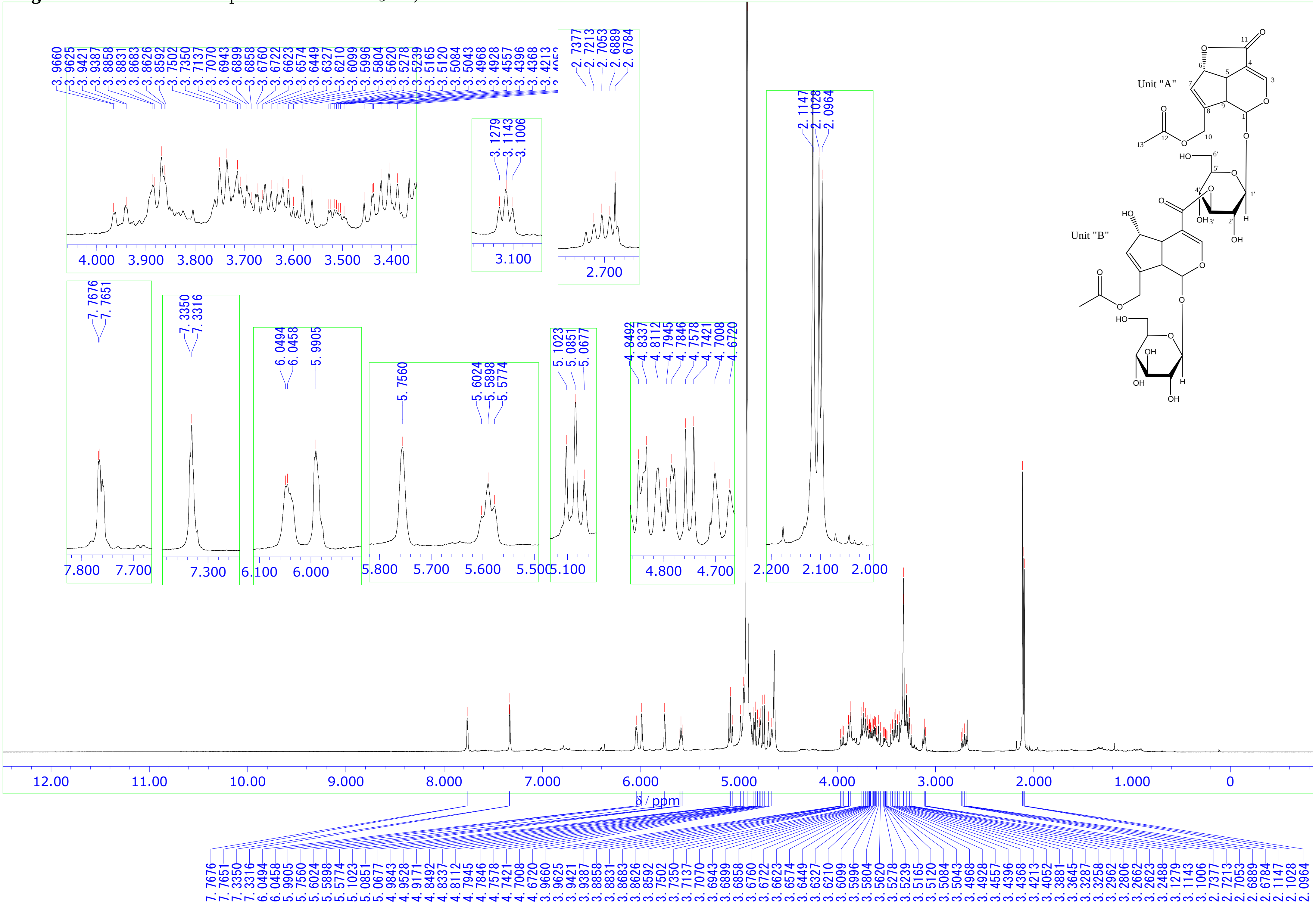

Figure S24. The ^{13}C NMR spectrum of **3** in CD_3OD, 175 MHz.

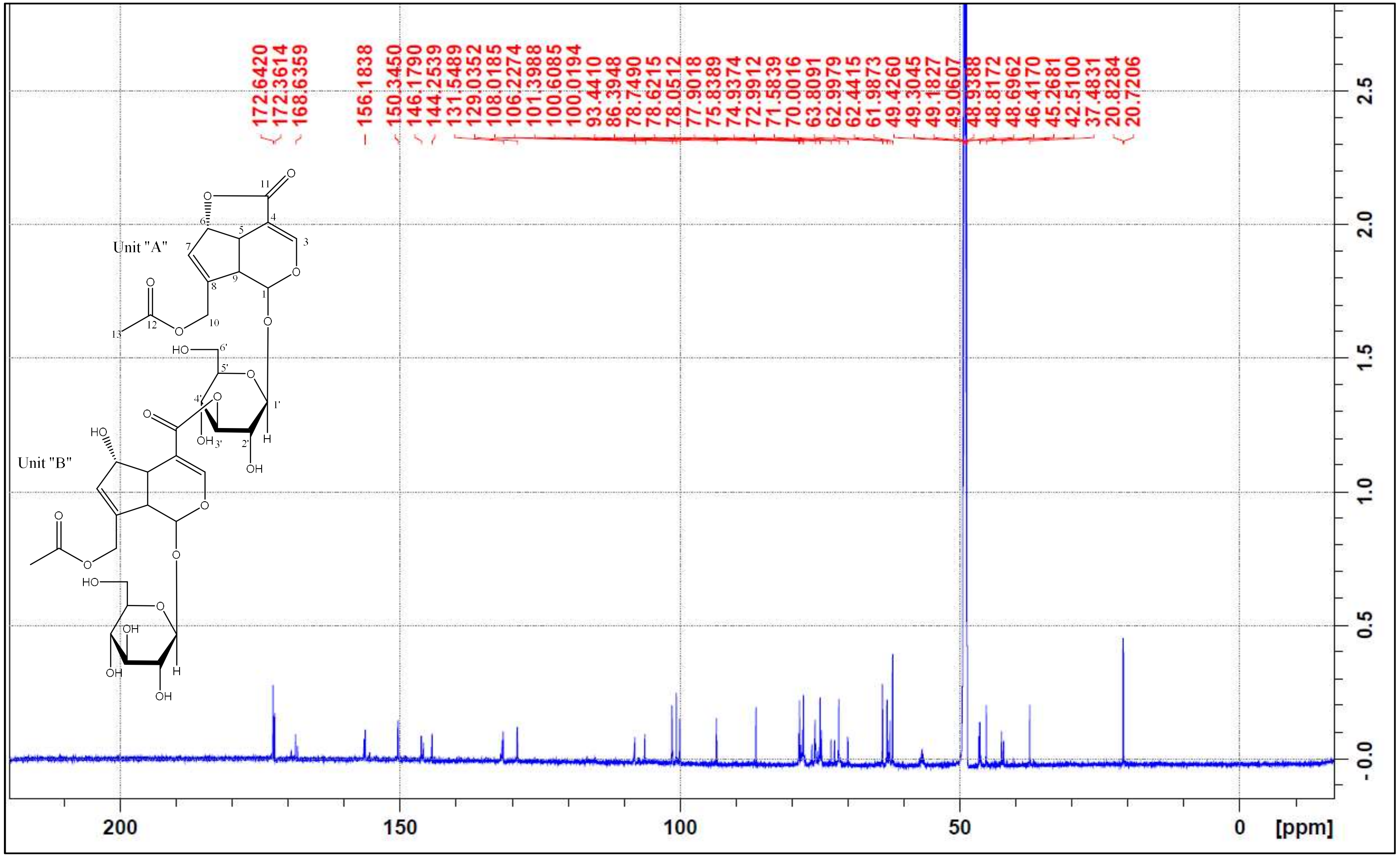

Figure S25. The DEPT-135 NMR spectrum of **3** in CD_3OD, 175 MHz.

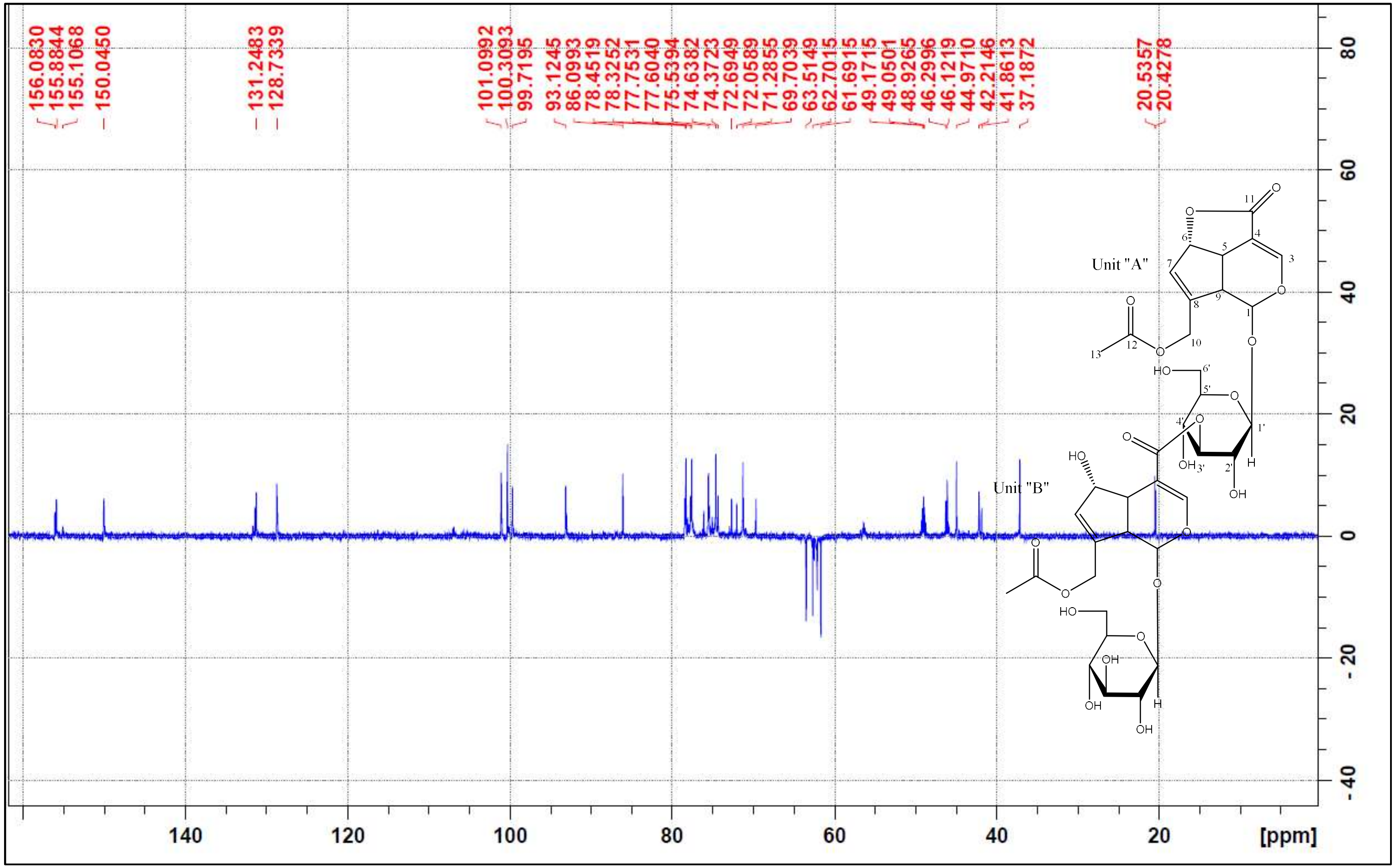

Figure S26. The COSY spectrum of **3** in CD_3OD, 500 MHz.

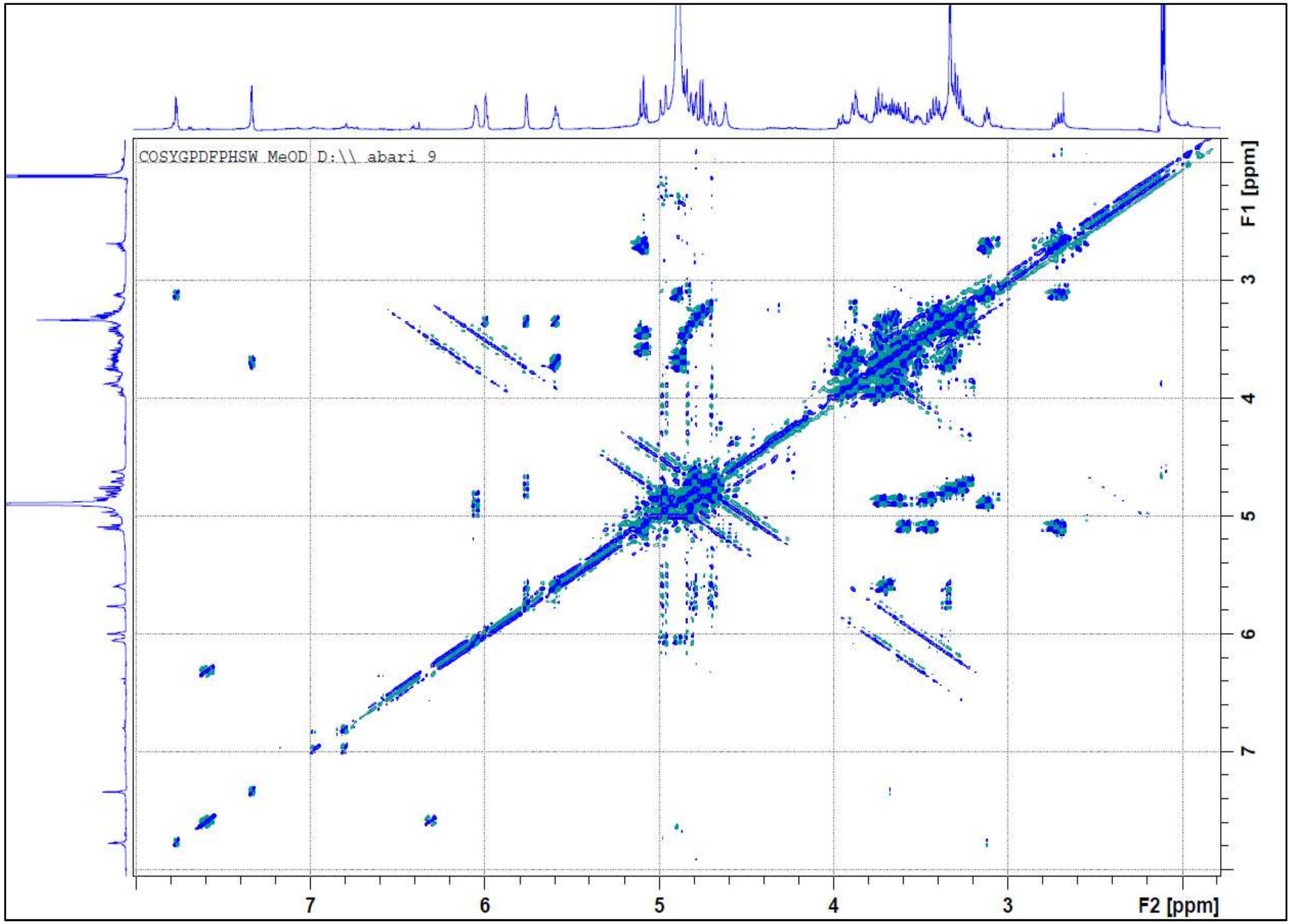

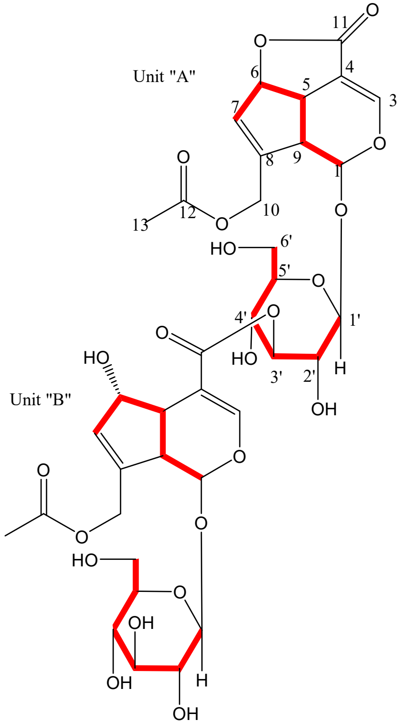

Figure S27. The HSQC spectrum of **3** in CD_3OD, 700 MHz.

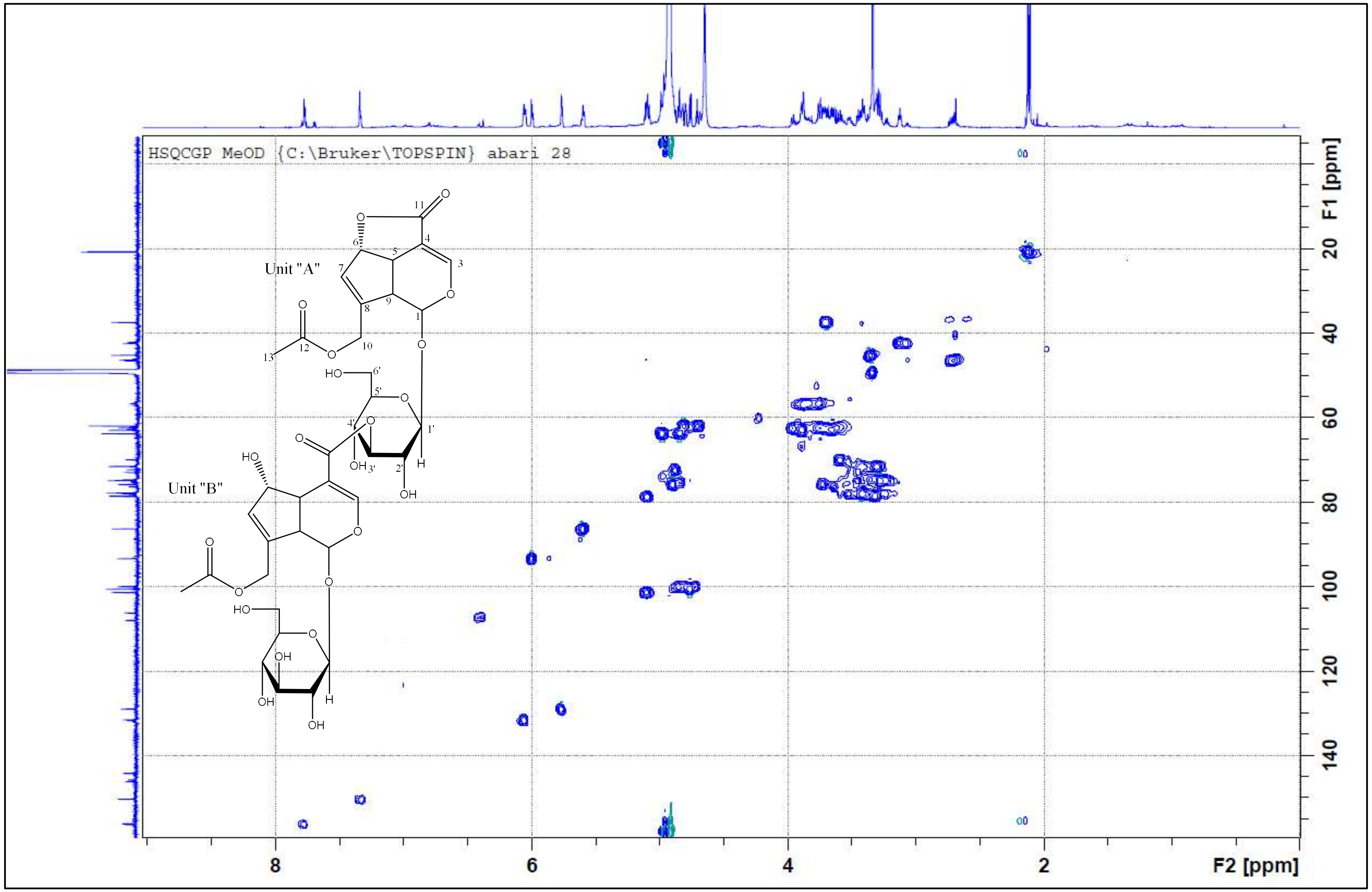

Figure S28. The HMBC spectrum of **3** in CD_3OD, 700 MHz.

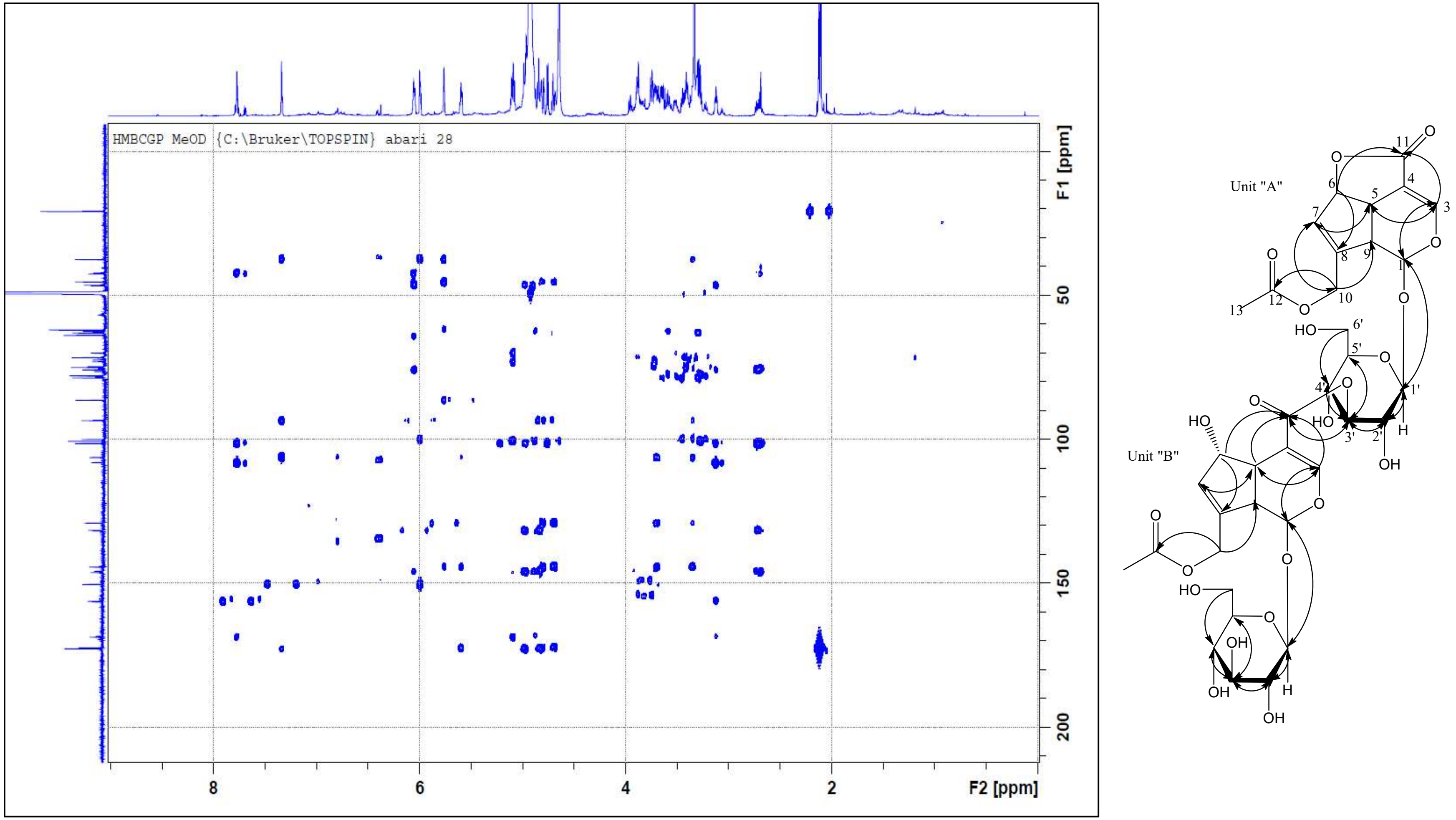

Figure S29. The NOESY spectrum of **3** in CD_3OD, 500 MHz.

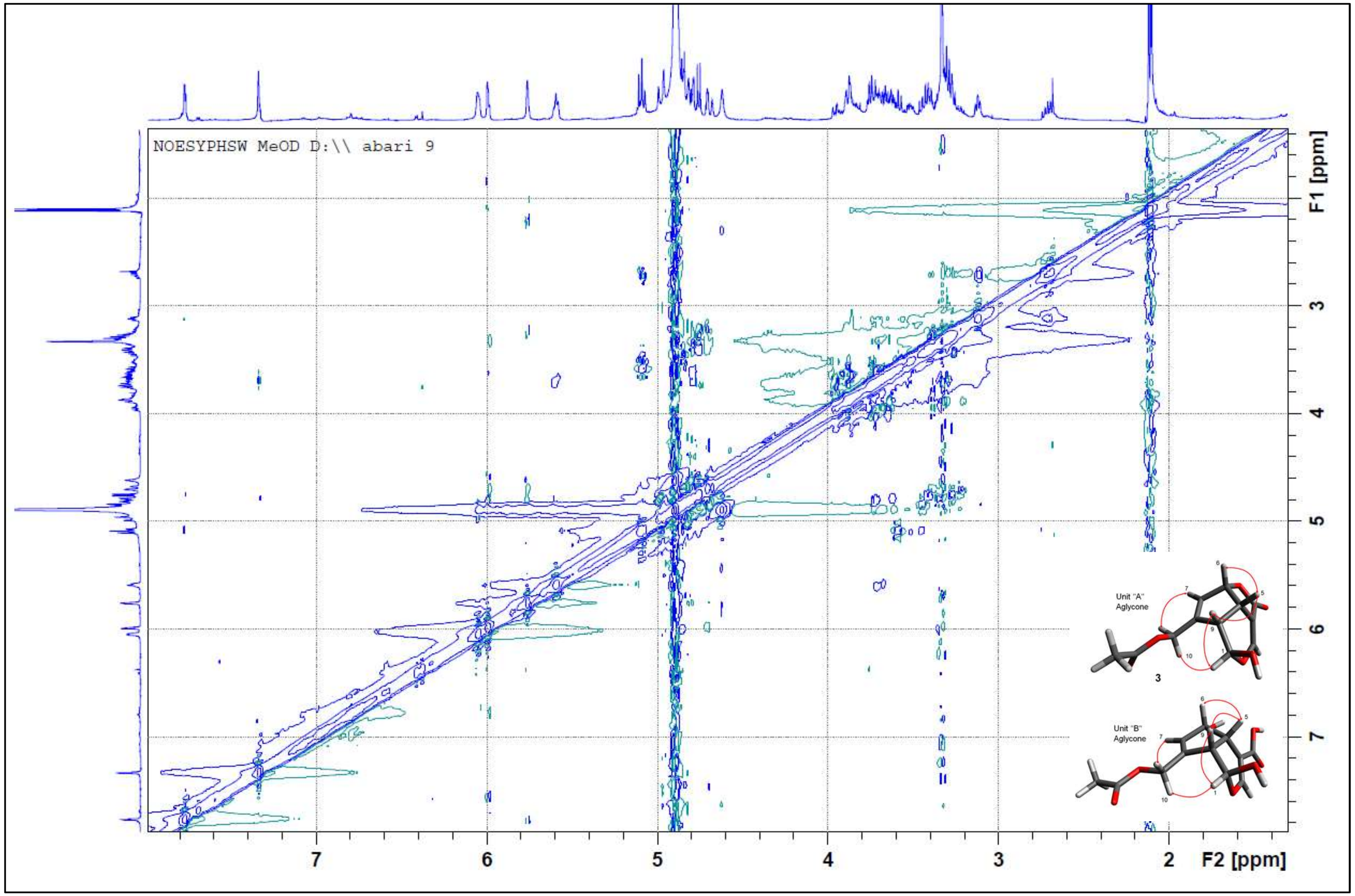

Figure S30. The UV spectrum of **3**.

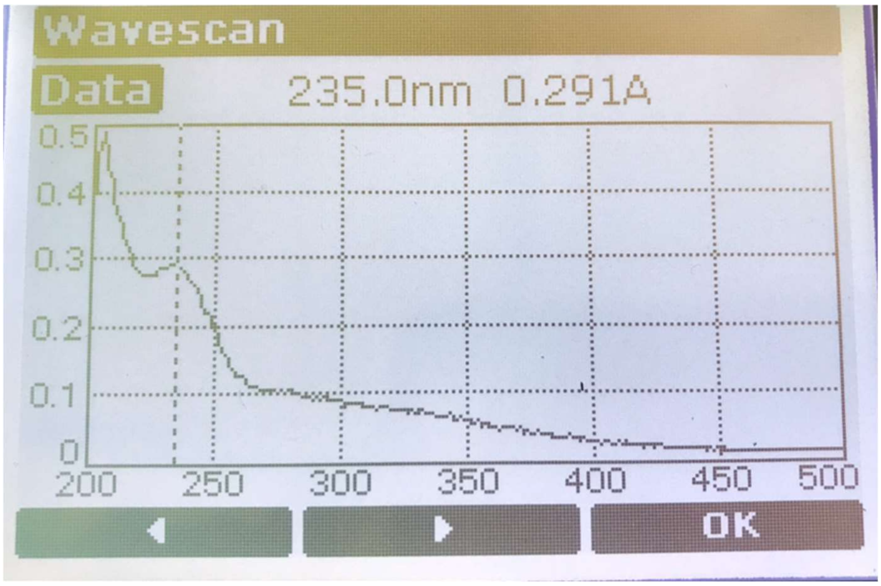

Figure S31. The IR spectrum of **3**.

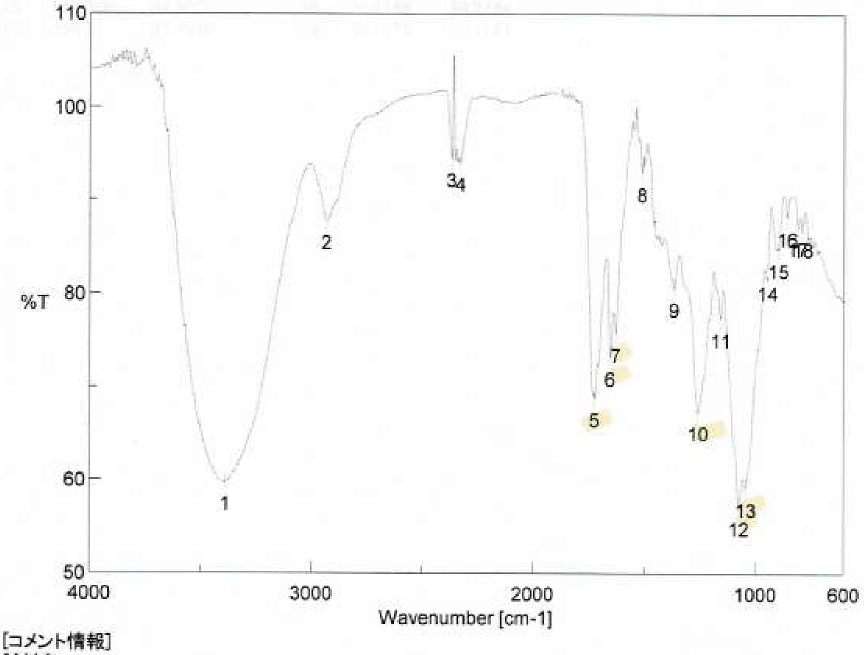

[コメント情報]

試料名	
コメント	
測定者	
所属	
会社	HU

[データ情報]

作成日時	2018/06/28 16:20
データタイプ	等間隔データ
横軸	Wavenumber [cm-1]
縦軸	%T
スタート	599.753 cm-1
エンド	7800.65 cm-1
データ間隔	0.964233 cm-1
データ数	7469

[測定情報]

機種名	FT/IR-4600typeA
シリアル番号	D015461786
測定日時	2018/06/28 16:20
光源	標準光源
検出器	TGS
積算回数	10
分解	4 cm-1
ゼロフィリング	On
アポダイゼーション	Cosine
ゲイン	Auto (2)
アパーチャー	Auto (7.1 mm)
スキャンスピード	Auto (2 mm/sec)
フィルタ	Auto (30000 Hz)

[ピーク検出結果]

No.	位置	強度	No.	位置	強度
1	3388.32	59.6747	2	2932.23	87.756
3	2368.16	94.4393	4	2327.66	93.9818
5	1729.83	68.8554	6	1658.48	73.1683
7	1632.45	75.6651	× 8	1513.85	92.8908
9	1369.21	80.5601	10	1261.22	67.4179
11	1158.04	77.2757	12	1075.12	57.1215
13	1044.26	59.1306	14	945.913	82.3818

[ピーク検出結果]

No.	位置	強度	No.	位置	強度
15	898.666	84.8025	16	858.168	88.2284
17	810.92	87.2016	18	787.779	87.1151

Figure S32. The HRESIMS spectrum of **4**.

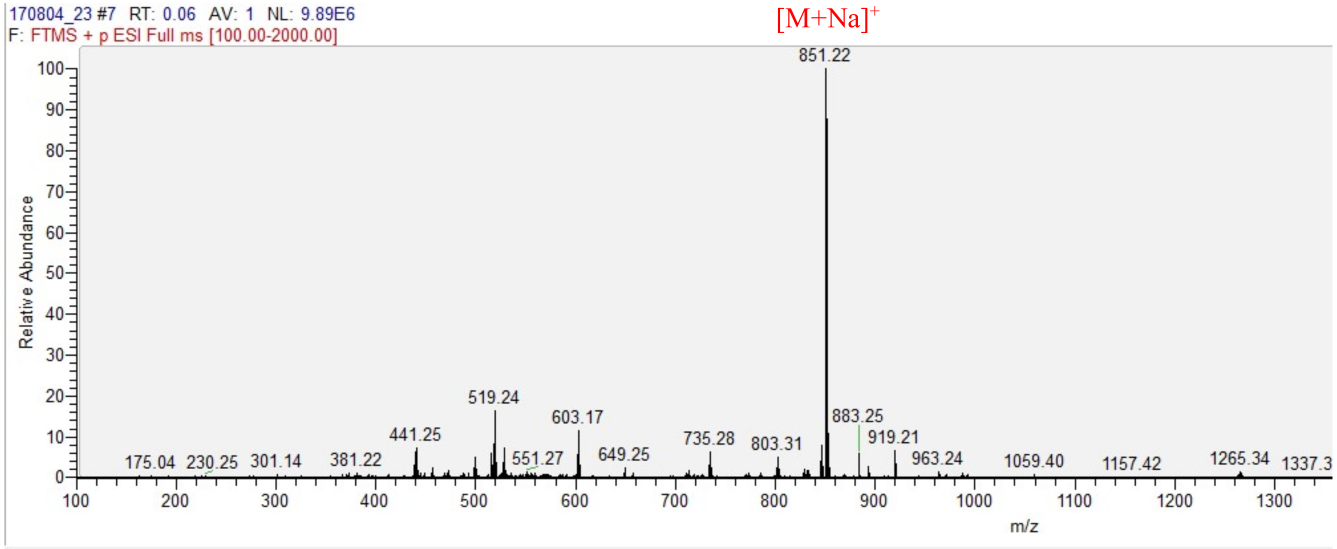

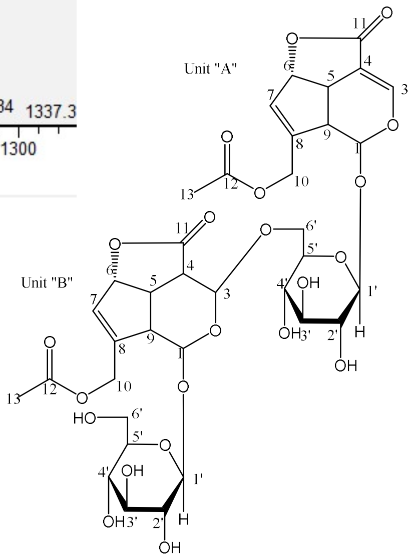

Elemental composition search on mass 851.22

m/z= 846.22-856.22

m/z	Theo. Mass	Delta (mmu)	RDB equiv.	Composition
851.2215	851.2216	-0.14	14.5	$C_{36} H_{44} O_{22} Na$

Figure S33. The ^{1}H NMR spectrum of **4** in CD_3OD, 500 MHz

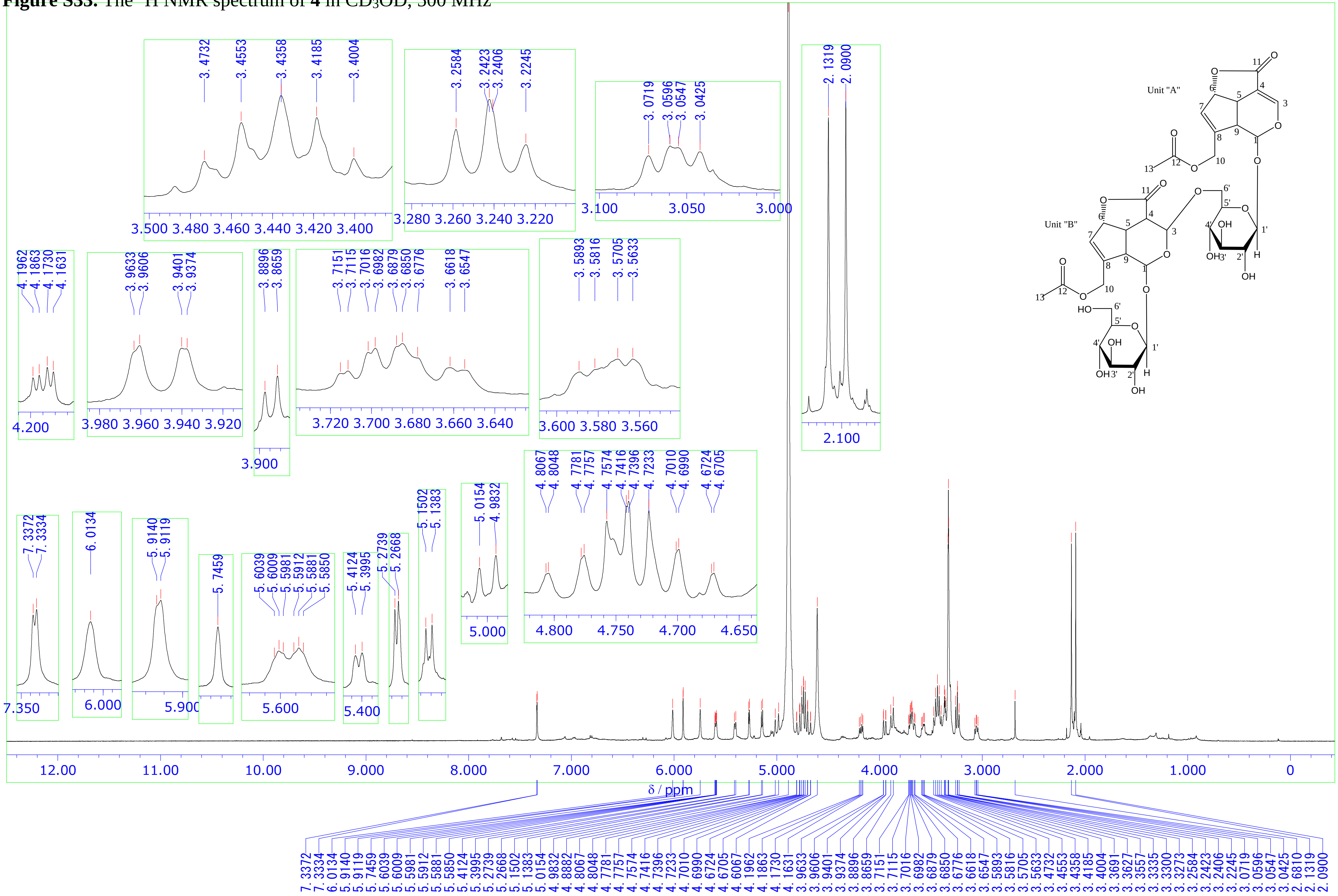

Figure S34. The ^{13}C NMR spectrum of **4** in CD_3OD, 175 MHz.

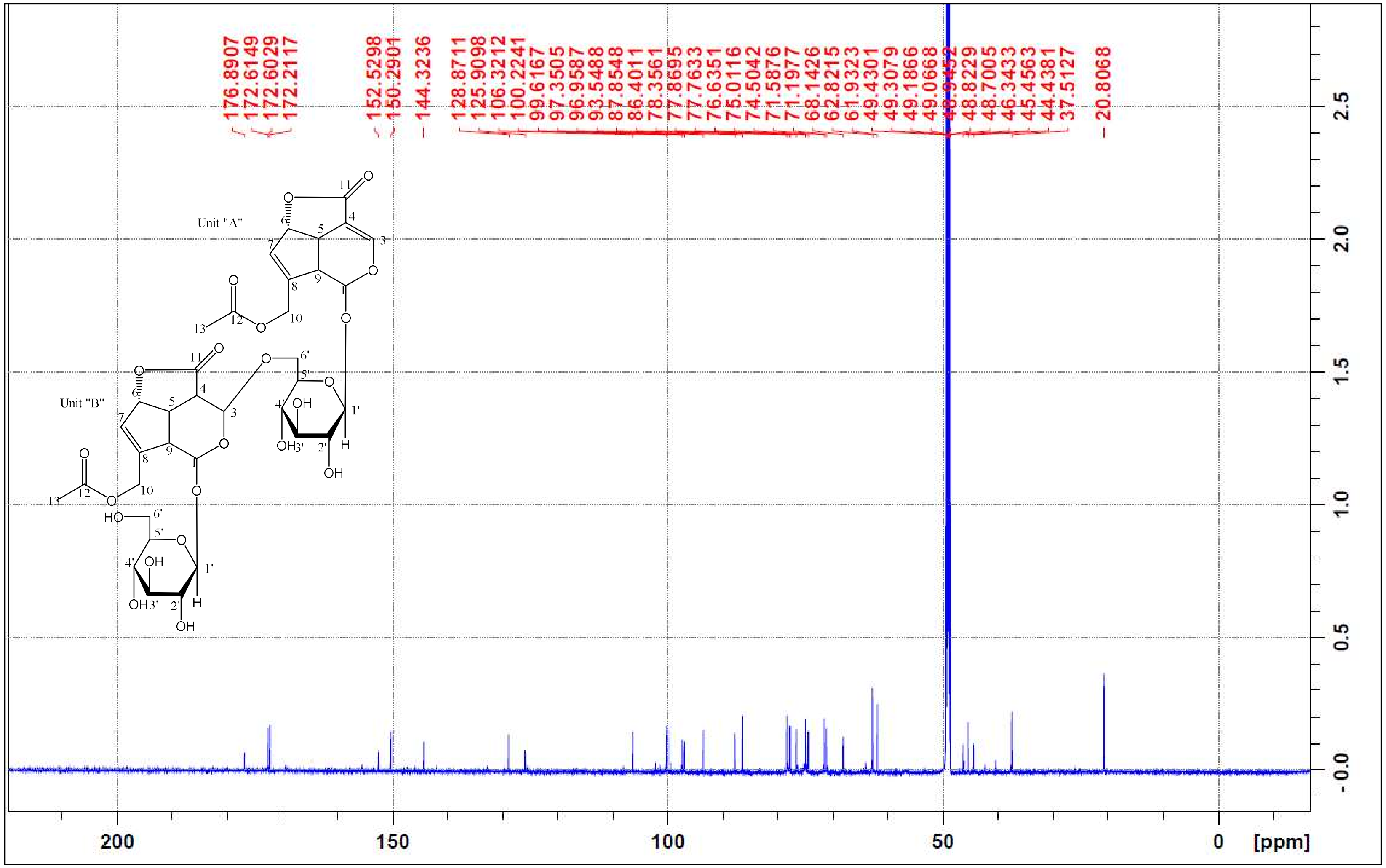

Figure S35. The DEPT-135 NMR spectrum of **4** in CD_3OD, 175 MHz

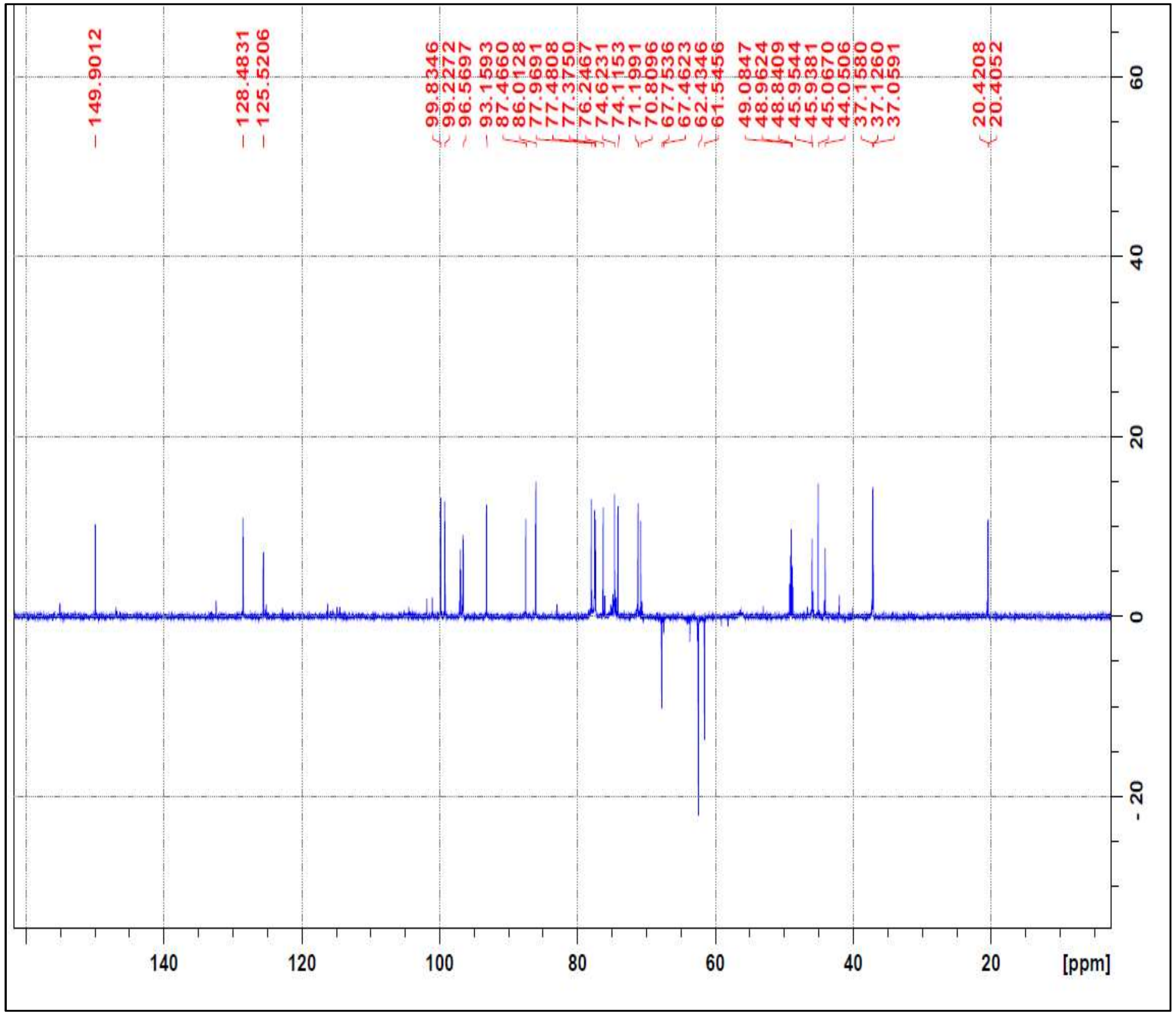

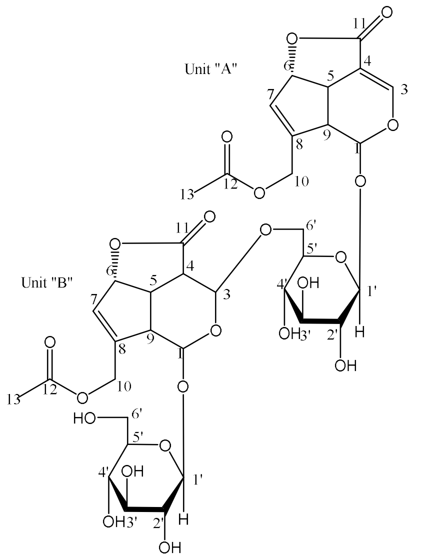

Figure S36. The COSY spectrum of **4** in CD_3OD, 500 MHz.

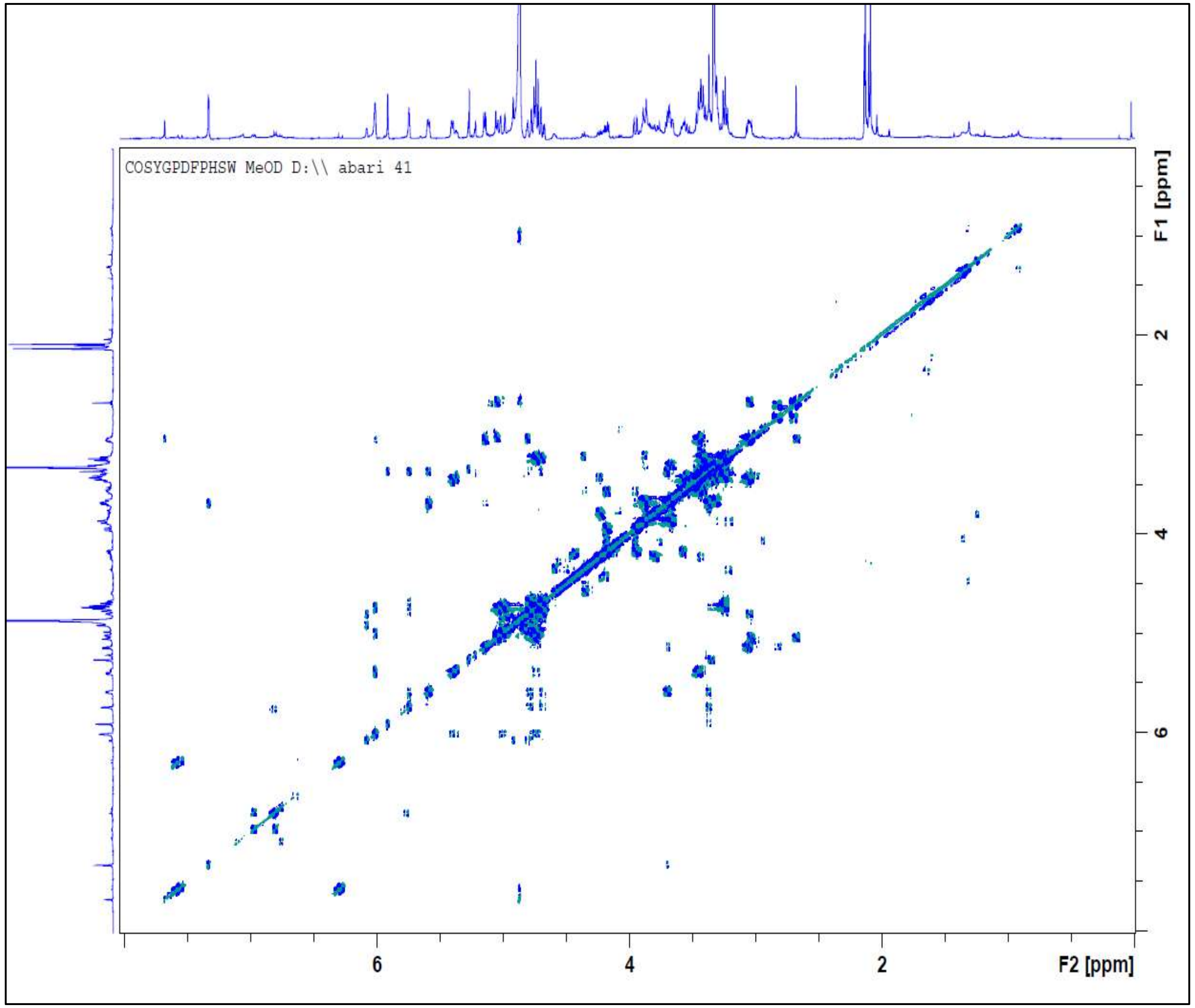

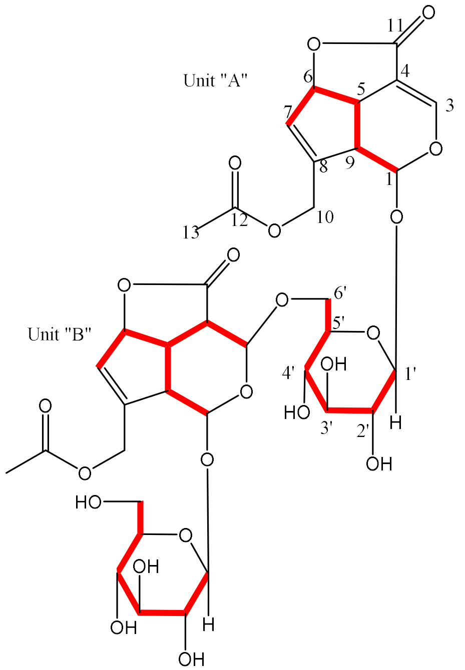

Figure S37. The HSQC spectrum of **4** in CD_3OD, 700 MHz

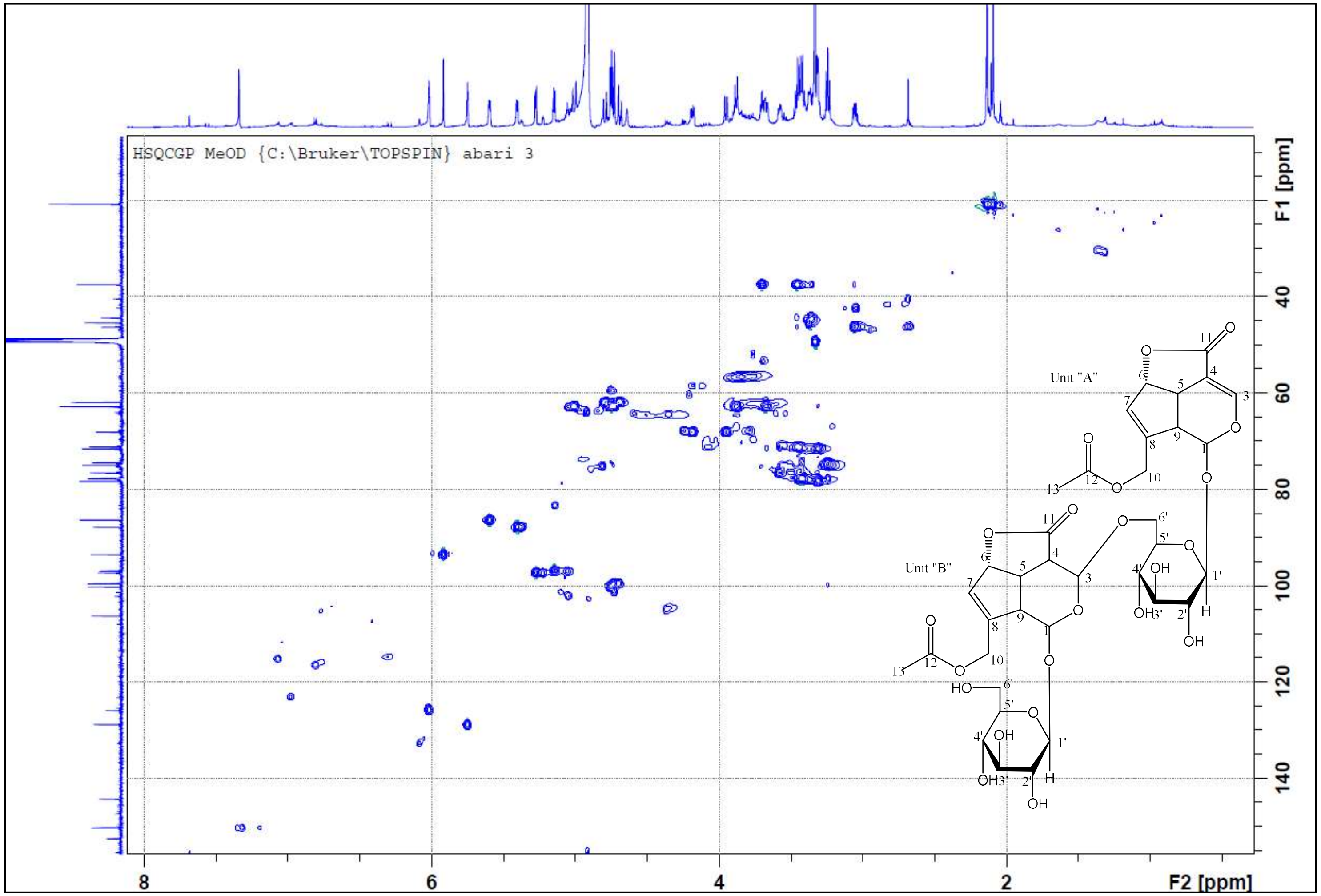

Figure S38. The HMBC spectrum of **4** in CD_3OD, 700 MHz

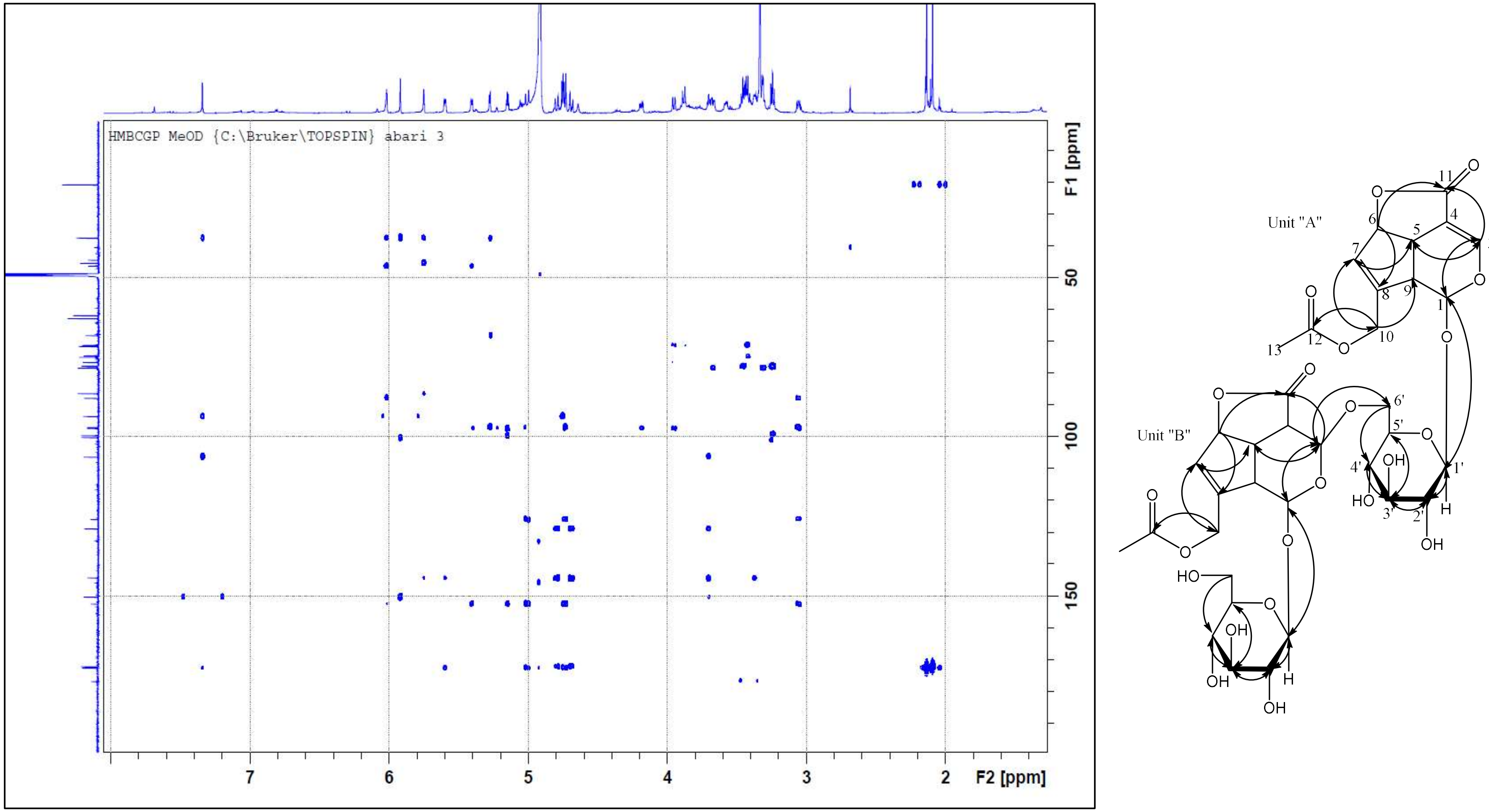

Figure S39. The NOESY spectrum of **4** in CD_3OD, 500 MHz .

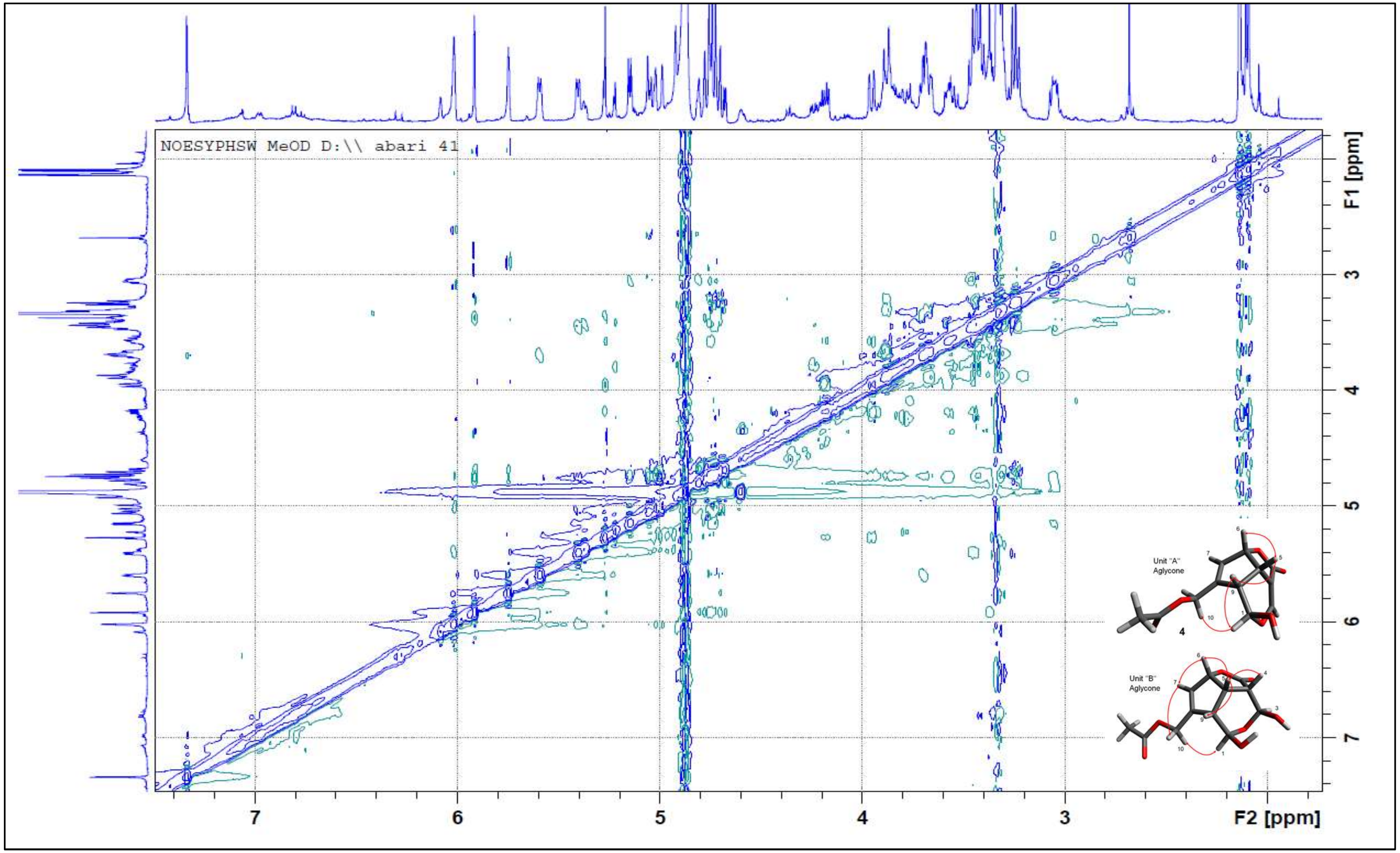

Figure S40. The UV spectrum of **4.**

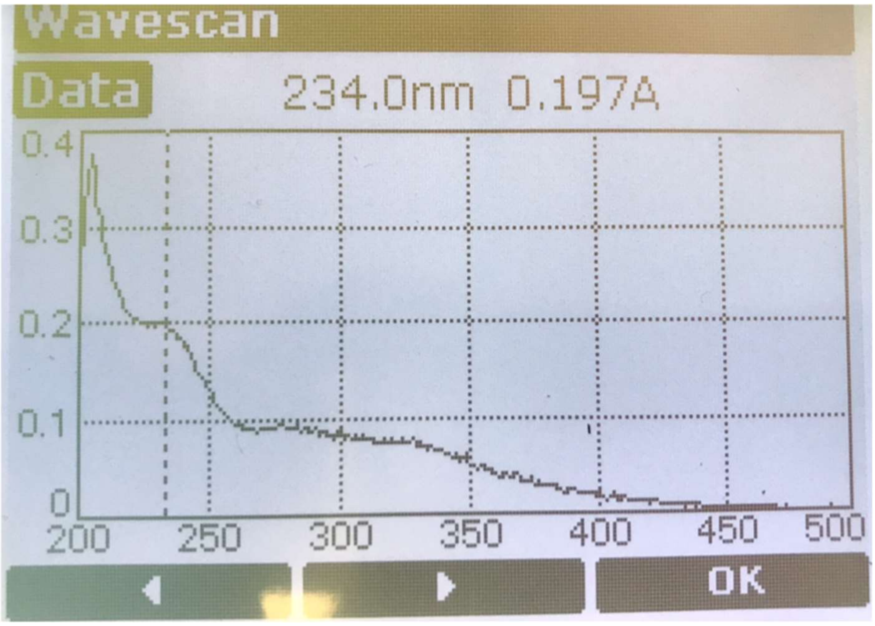

Figure S41. The IR spectrum of **4**.

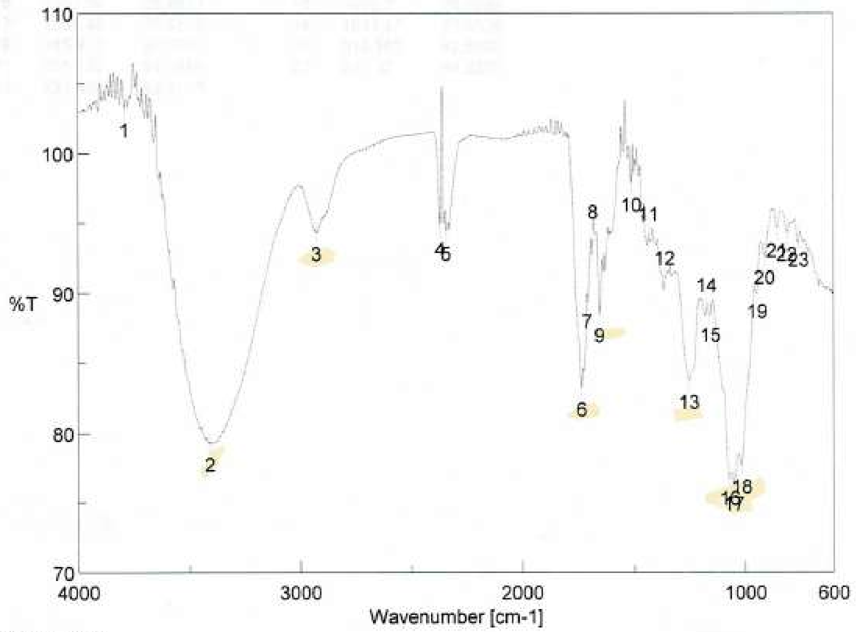

[コメント情報]
試料名
コメント
測定者
所属
会社 HU

[データ情報]		[測定情報]	
作成日時	2018/06/28 16:12	機種名	FT/IR-4600typeA
		シリアル番号	D015461786
データタイプ	等間隔データ		
横軸	Wavenumber [cm-1]	測定日時	2018/06/28 16:12
縦軸	%T		
スタート	599.753 cm-1	光源	標準光源
エンド	7800.65 cm-1	検出器	TGS
データ間隔	0.964233 cm-1	積算回数	10
データ数	7469	分解	4 cm-1
		ゼロフィリング	On
		アポダイゼーション	Cosine
		ゲイン	Auto (2)
		アパーチャー	Auto (7.1 mm)
		スキャンスピード	Auto (2 mm/sec)
		フィルタ	Auto (30000 Hz)

[ピーク検出結果]

No.	位置	強度	No.	位置	強度
1	3790.4	103.21	2	3406.64	79.2507
3	2927.41	94.3485	4	2369.12	94.6634
5	2341.16	94.3638	6	1738.51	83.2329
7	1713.44	89.4801	8	1687.41	94.1658
9	1658.48	88.4395	10	1513.85	97.8128
11	1434.78	94.0398	12	1363.43	90.9109
13	1254.47	83.75	14	1175.4	88.939

ク検出結果]

	位置	強度	No.	位置	強度
15	1157.08	88.4817	16	1070.3	76.8286
17	1051.98	76.4215	18	1017.27	77.6526
19	945.913	90.1789	20	912.165	92.6303
21	856.239	94.5814	22	810.92	94.3202
23	757.888	93.9102			
