# Supplementary material for: Lasianosides F–I: A New Iridoid and Three New Bis-Iridoid Glycosides from the Leaves of Lasianthus verticillatus (Lour.) Merr
Source: Molecules. 2020 Jun 17;25(12):2798. doi: 10.3390/molecules25122798 (PMC7356845; doi:10.3390/molecules25122798)
Supplement: Supplementary file 1 [file molecules-25-02798-s001.zip › Supplementary file/NMR data.zip!/NMR data/Cpd 1 13C/email_drHamoud-113E-1_12_1.pdf]

C13CPD MeOD {C:\Bruker\TOPSPIN} abari 5

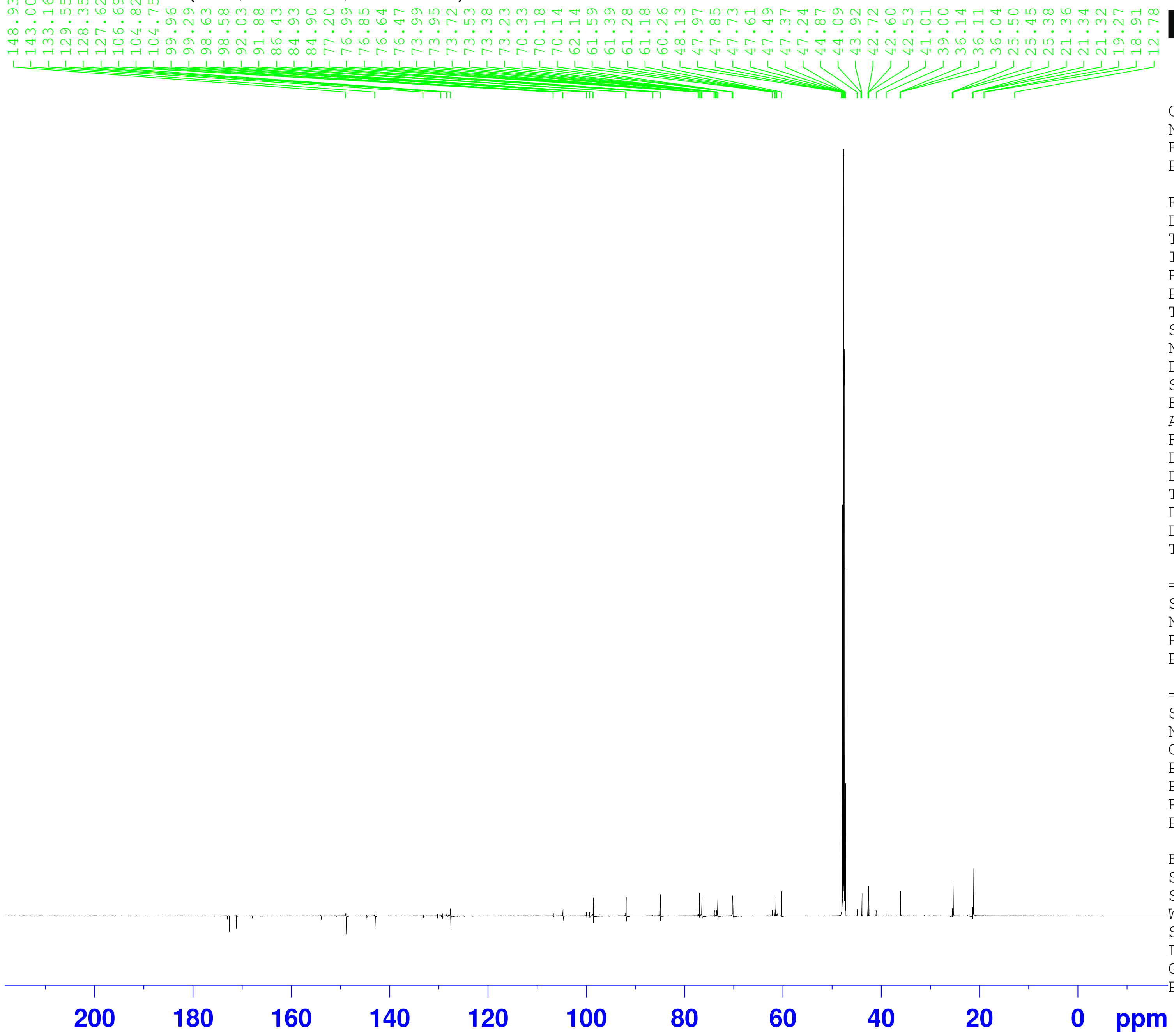

Current Data Parameters

NAME	drHamoud-113E-1
EXPNO	12
PROCNO	1

F2 - Acquisition Parameters

Date_	20171022
Time	22.42
INSTRUM	spect
PROBHD	5 mm CPTCI 1H-
PULPROG	zgpg30
TD	65536
SOLVENT	MeOD
NS	4000
DS	4
SWH	41666.668 Hz
FIDRES	0.635783 Hz
AQ	0.7864320 sec
RG	172.3
DW	12.000 usec
DE	18.00 usec
TE	295.0 K
D1	2.00000000 sec
D11	0.03000000 sec
TD0	1

======== CHANNEL f1 ========

SFO1	176.0754915 MHz
NUC1	13C
P1	12.00 usec
PLW1	121.00000000 W

======== CHANNEL f2 ========

SFO2	700.1728007 MHz
NUC2	1H
CPDPRG[2	waltz16
PCPD2	65.00 usec
PLW2	9.64999962 W
PLW12	0.14618000 W
PLW13	0.06176000 W

F2 - Processing parameters

SI	32768
SF	176.0578870 MHz
WDW	EM
SSB	0
LB	1.00 Hz
GB	0
PC	1.40
