# Supplementary material for: Lasianosides F–I: A New Iridoid and Three New Bis-Iridoid Glycosides from the Leaves of Lasianthus verticillatus (Lour.) Merr
Source: Molecules. 2020 Jun 17;25(12):2798. doi: 10.3390/molecules25122798 (PMC7356845; doi:10.3390/molecules25122798)
Supplement: Supplementary file 1 [file molecules-25-02798-s001.zip › Supplementary file/NMR data.zip!/NMR data/Cpd 4 13C/email_drHamoud-113-G2_11_1.pdf]

C13CPD MeOD {C:\Bruker\TOPSPIN} abari 3

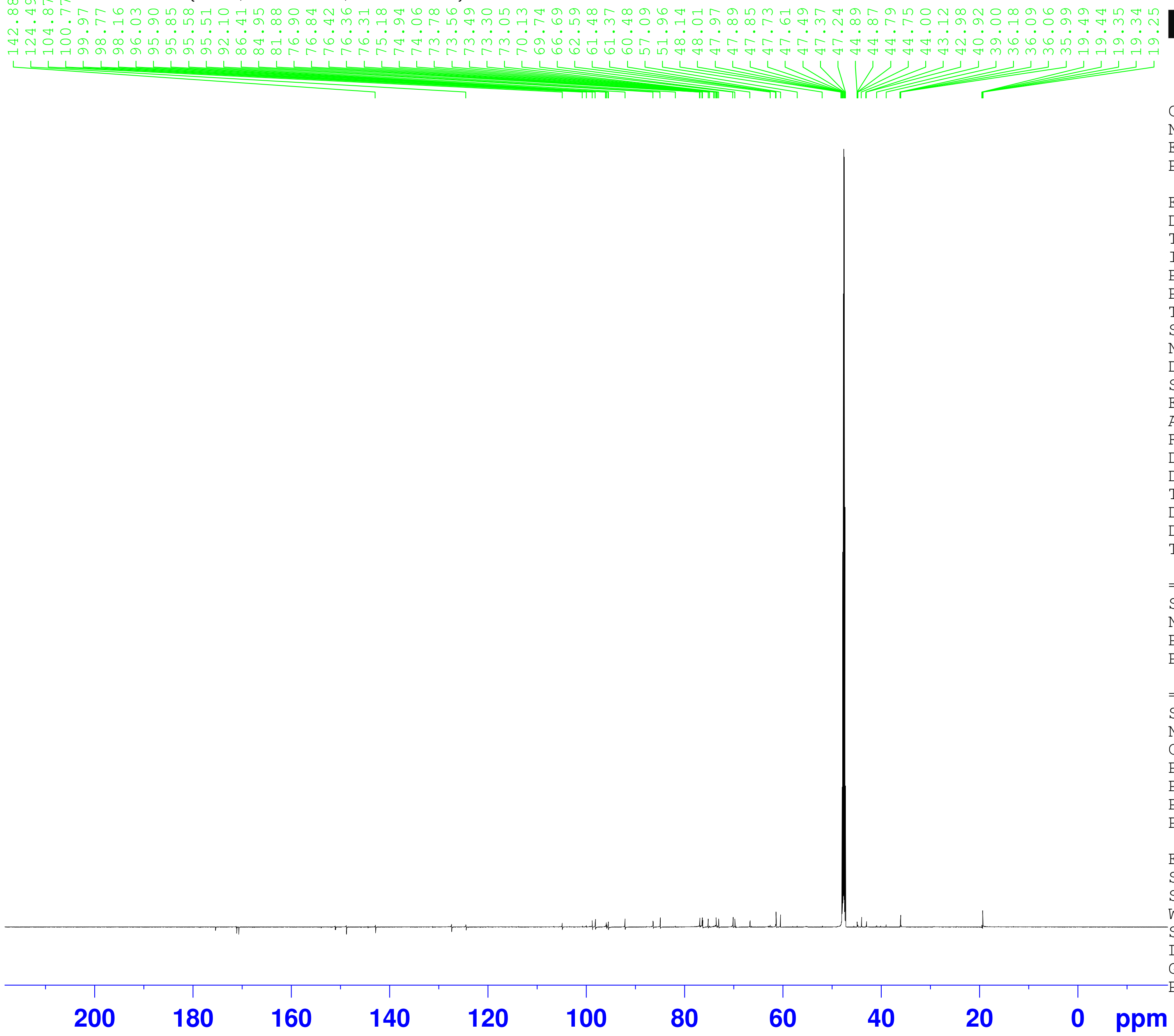

Current Data Parameters
NAME drHamoud-113-G2
EXPNO 11
PROCNO 1

F2 - Acquisition Parameters
Date_ 20171225
Time 3.39
INSTRUM spect
PROBHD 5 mm CPTCI 1H-
PULPROG zgpg30
TD 65536
SOLVENT MeOD
NS 3000
DS 4
SWH 41666.668 Hz
FIDRES 0.635783 Hz
AQ 0.7864320 sec
RG 172.3
DW 12.000 usec
DE 18.00 usec
TE 295.0 K
D1 2.00000000 sec
D11 0.03000000 sec
TD0 1

======== CHANNEL f1 ========
SFO1 176.0754915 MHz
NUC1 13C
P1 12.00 usec
PLW1 121.00000000 W

======== CHANNEL f2 ========
SFO2 700.1728007 MHz
NUC2 1H
CPDPRG[2 waltz16
PCPD2 65.00 usec
PLW2 9.64999962 W
PLW12 0.14618000 W
PLW13 0.06176000 W

F2 - Processing parameters
SI 32768
SF 176.0578870 MHz
WDW EM
SSB 0
LB 1.00 Hz
GB 0
PC 1.40
